# The Biosynthesis of Rare Homo-Amino Acid Containing Variants of Microcystin by a Benthic Cyanobacterium

**DOI:** 10.3390/md17050271

**Published:** 2019-05-07

**Authors:** Tânia Keiko Shishido, Jouni Jokela, Anu Humisto, Suvi Suurnäkki, Matti Wahlsten, Danillo O. Alvarenga, Kaarina Sivonen, David P. Fewer

**Affiliations:** 1 Department of Microbiology, University of Helsinki, Viikinkaari 9, FI-00014 Helsinki, Finland; tania.shishido@helsinki.fi (T.K.S.); jouni.jokela@helsinki.fi (J.J.); anu.humisto@helsinki.fi (A.H.); suvi.a.e.suurnakki@jyu.fi (S.S.); matti.wahlsten@helsinki.fi (M.W.); danillo.oliveiradealvarenga@helsinki.fi (D.O.A.); kaarina.sivonen@helsinki.fi (K.S.); 2 Institute of Biotechnology, University of Helsinki, Viikinkaari 5D, FI-00014 Helsinki, Finland; 3 Department of Biological and Environmental Science, University of Jyväskylä, FI-40014 Jyväskylä, Finland

**Keywords:** adenylation domain, hepatotoxin, cyanobacteria, nonribosomal peptide synthetase (NRPS), polyketide synthase (PKS), mass spectrometry

## Abstract

Microcystins are a family of chemically diverse hepatotoxins produced by distantly related cyanobacteria and are potent inhibitors of eukaryotic protein phosphatases 1 and 2A. Here we provide evidence for the biosynthesis of rare variants of microcystin that contain a selection of homo-amino acids by the benthic cyanobacterium *Phormidium* sp. LP904c. This strain produces at least 16 microcystin chemical variants many of which contain homophenylalanine or homotyrosine. We retrieved the complete 54.2 kb microcystin (*mcy*) gene cluster from a draft genome assembly. Analysis of the substrate specificity of McyB_1_ and McyC adenylation domain binding pockets revealed divergent substrate specificity sequences, which could explain the activation of homo-amino acids which were present in 31% of the microcystins detected and included variants such as MC-LHty, MC-HphHty, MC-LHph and MC-HphHph. The *mcy* gene cluster did not encode enzymes for the synthesis of homo-amino acids but may instead activate homo-amino acids produced during the synthesis of anabaenopeptins. We observed the loss of microcystin during cultivation of a closely related strain, *Phormidium* sp. DVL1003c. This study increases the knowledge of benthic cyanobacterial strains that produce microcystin variants and broadens the structural diversity of known microcystins.

## 1. Introduction

Microcystins are potent hepatotoxins, which inhibit eukaryotic protein phosphatases type 1 and 2A [1]. Microcystins are frequently detected during blooms of cyanobacteria in freshwater environments and are linked to poisoning of humans and animals [2,3]. Microcystins have an unusual chemical structure with more than 240 reported chemical variants [4]. The microcystin chemical structure can be summarized as cyclo-D-Ala^1^-X^2^-D-MeAsp^3^-Z^4^-Adda^5^-D-Glu^6^-Mdha^7^ where X and Z are variable L-amino acids. The bulk of microcystin chemical variation can be attributed to the amino acids found at positions two (X) and four (Z).

Microcystins are synthesized by large hybrid multifunctional enzymes known as nonribosomal peptide synthetases (NRPS) and polyketide synthases (PKS) [5,6,7,8]. The chemical variability of microcystins is mainly attributed to a substrate promiscuity of microcystin biosynthetic enzymes, which allows the incorporation of a diverse range of proteinogenic/non-proteinogenic amino acids, non-proteinogenic organic acids and different degrees of methylation [9]. This is due to differences in substrate specificity of the NRPS enzymes, the deletion of enzymatic domains or loss/gain of tailoring enzymes [1,8].

Homo-amino acids have an extra methylene (CH_2_) group in the carbon side chain and are frequently found in cyanobacterial natural products [9,10,11,12,13,14,15,16]. Cyanobacteria have been found to produce microcystins that contain homo-amino acids in positions X and Z, such as homotyrosine (Hty), homophenylalanine (Hph), homoisoleucine (Hil), and homoarginine (Har) [4,10,11,12,13]. However, the biosynthetic origins of such microcystins are unclear.

*Phormidium* is a genus of cyanobacteria belonging to Oscillatoriales order that includes unbranched filamentous strains. *Phormidium* is not well defined taxonomically and further studies are needed to improve its separation with other genera of Oscillatoriales [17], which complicates the interpretation of reports about *Phormidium* in the scientific literature. Cyanobacteria belonging to *Phormidium* genus have been isolated from mangrove [18], saline-alkaline lakes [19], thermal springs [20], high altitude lakes [21] and mats/biofilms in Antarctic lakes [22] among others. *Phormidium* spp. isolated from Egypt, USA, Brazil and Spain have been reported to produce microcystins [13,23,24,25]. Putative *Phormidium* spp. producers of microcystin were reported to be associated to the poisoning of a dog in New Zealand [26]. However, analysis based on 16S rRNA sequences indicates a close similarity of these strains with *Planktothrix* [26]. Microcystin-LR was detected from *Phormidium* strains isolated from benthic environments in USA [24] and here we further analyze selected strains from that study. We describe the microcystin biosynthetic gene cluster from *Phormidium* sp. LP904c and show that this strain produces rare homo-amino acid containing microcystins in addition to microcystin-LR. We also report the loss of the microcystin synthesis by *Phormidium* sp. DVL1003c after 11 years of cultivation in our laboratory.

## 2. Results

### 2.1. Phormidium Strains Producing Unusual Microcystins (MCs)

**Figure 1:**
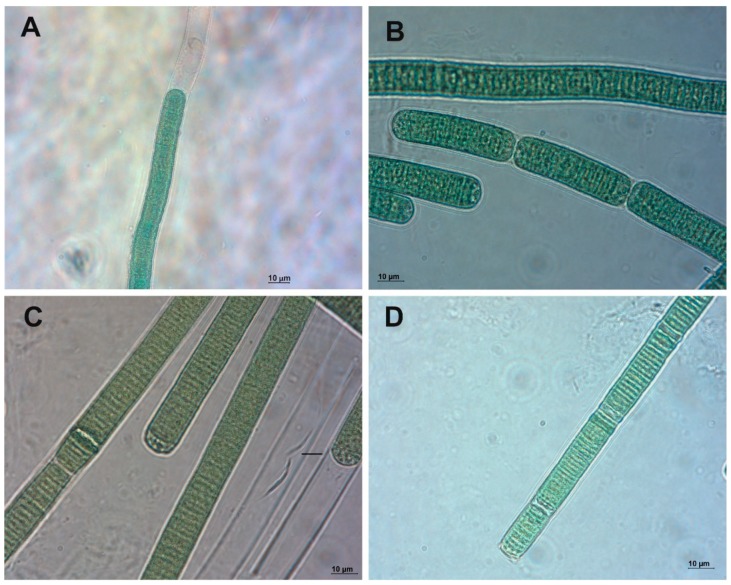
Photomicrography of the studied *Phormidium* strains. (**A**) LP904c. (**B**) DVL1003c. (**C**) LM603d. (**D**) LS703b.

**Figure 2:**
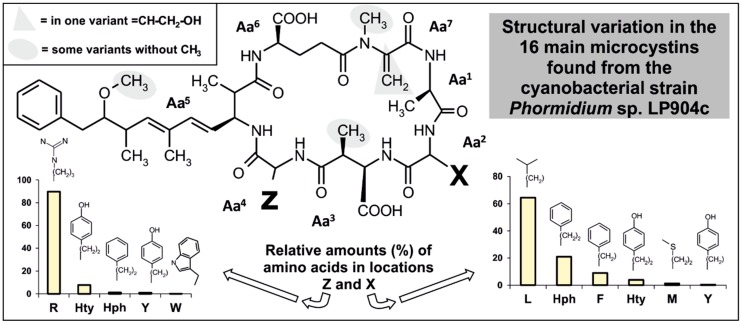
Structural variation in the 16 microcystins detected in the *Phormidium* sp. LP904c. The column charts indicate the relative amounts of amino acids (%) detected in the X and Z positions of microcystins. Hty = homotyrosine, Hph = homophenylalanine.

*Phormidium* sp. LP904c is a benthic cyanobacterium isolated from the Lake Perris in Riverside County, California (USA) that is reported to produce microcystin-LR. This cyanobacterium was found to produce diverse microcystins varying in the positions X and Z (Figure 1A). Further variations were related to the presence or absence of methylation in the aspartic acid (Aa^3^) or Adda (Aa^5^) and for the presence of a methylated serine or dehydroalanine instead of the N-methyldehydroalanine (Aa^7^) (Figure 2).

MC-LR was the main variant as previously reported [24] and accounted for 55% of the total microcystin produced by this strain. However, the strain produces a range of other microcystins including MC-LHty, MC-HphHty, MC-LHph and MC-HphHph (Figure 2, Table 1). A number of amino acids were found in position X and Z, such as Leu, Arg, Phe, Trp, Tyr, Met and unusual homo-amino acids such as Hph and Hty (Figure 2, Table 1).

*Phormidium* sp. DVL1003c (Figure 1B) was isolated from a freshwater reservoir in Riverside County, California. Strains DVL1003c and LP904c were shown to synthesize similar microcystin variants in 2005 (Table 1, Supplementary Figures S1–S3). Structural characterizations of microcystins produced by *Phormidium* sp. LP904c were done using high-performance liquid chromatography/electrospray ionization ion-trap mass spectrometry (HPLC-ESI-ITMS) and ultra-high performance liquid chromatography-quadrupole time-of-flight mass spectrometry (UPLC-QTOF). MCs identified were divided to two groups, ten arginine (Arg) containing MCs (Numbers 1–10) and six non Arg MCs (Numbers 11–16) (Table 1). All Arg MCs eluted first, followed by the more hydrophobic non Arg MCs (Supplementary Figures S2 and S3). These two MC groups fragment differently as protonated molecules resulting in distinct product ion spectra (Supplementary Figure S4). Both spectral groups were highly similar to spectra recorded in similar ion trap conditions [27]. In Arg containing MC structures fragmentation prefers the formation of ion 4a–6c (relative intensities (RI) from 51 to 100%, Arg^4^-Adda^5^-Glu^6^, Supplementary Figures S4 and S5) which have an *m*/*z* 599 when methyl group 2 is present. When the methyl group is absent, e.g., [DMAdda^5^]MC, an *m*/*z* 585 is detected. Serine in MC structure generates a base peak of [M + H − H_2_O]^+^ which was seen in the spectra of [MeSer^7^]MC-LR and which lowered the relative intensity of 4a-6c ion (Supplementary Figure S4). Ions 4a-6c – CO (RI 26–94%) and 4a–6c – NH_3_ (RI 6–28%) were also present (Supplementary Figure S4). The presence of ion 4a–7c (RI 4–20%, Arg^4^-Adda^5^-Glu^6^-Aa^7^, Supplementary Figure S5) shows the identity of the Aa^7^. Other diagnostic intense ions were 4a-2c/7a-5c (RI 8–38%) which showed the identity of amino acids 3 and 6, and 7a–4c (RI 45–94%, Aa^7^-Ala^1^-Aa^2^-MeAsp^3^-Arg^4^) together with other ions showed the identity of amino acids 4 and 5. Ion 5y-5x-7c was present in all the protonated MC product ion spectra except in the [DMAdda^5^]MC-LR spectra because cleavage at position 5y is not so favorable when there is hydroxyl group instead of the methoxy group vicinal to the 5y bond [28]. In the presence of methyl group 3 the ion mass is *m*/*z* 375 or 393 when Aa^7^ is MeSer and when it is absent the mass is *m*/*z* 361, such as in the [Dha^7^]MC-LR. Ion 5y-5x-7c identifies amino acids 5, 6 and 7. Identification of amino acids 1 and 2 using MS is challenging because the intensities of the diagnostic ions are near the noise level but for example ion 4a–1c (*m*/*z* 753) was clearly present in the spectrum of MC-MR showing the presence of methionine in this microcystin (Supplementary Figures S4 and S5).

The base peak is [M + H − C_9_H_10_O]^+^ in the product ion spectra of protonated non Arg microcystins [29]. In the *Phormidium* sp. LP904c microcystins spectra, the most intense peak was [M + H − NH_3_]^+^ followed by [M + H − C_9_H_10_O]^+^ (same as 5y), [M + H − (NH_3_ + C_9_H_10_O)]^+^ (same as 5y-5x-4c) and [M + H − H_2_O]^+^ (Supplementary Table S1). The ion 5y-5x-7c was present in all protonated MC product ion spectra identifying amino acids 5, 6 and 7 (Supplementary Figure S4). Medium intensity ions 5y-5x-1c (*m*/*z* 446) and 5y-5x-2c, which are present in all non Arg MCs, identify the amino acids 1 and 2. Ions 5y-5x-3c and 5y-5x-4c identify amino acids 3 and 4. The low intensity of ion 5y-5x-3c in some MCs weakened the certainty of amino acids 2 and 4. Product ion spectra of sodiated non-Arg microcystins are presented in Supplementary Figure S6 and product ion assignments are presented in Supplementary Table S2. These data support the assignments of the protonated product ions but do not clearly enhance the identity of amino acids 2 and 4. Additional proof for the MC structures gave the accurate masses measured from the protonated molecules error being lower than ±2.4 ppm (Table 1). The immonium and other diagnostic ions of amino acids 2, 4 measured with UPLC-QTOF were also in agreement with the presented MC structures (Supplementary Table S3).

**Figure 3:**
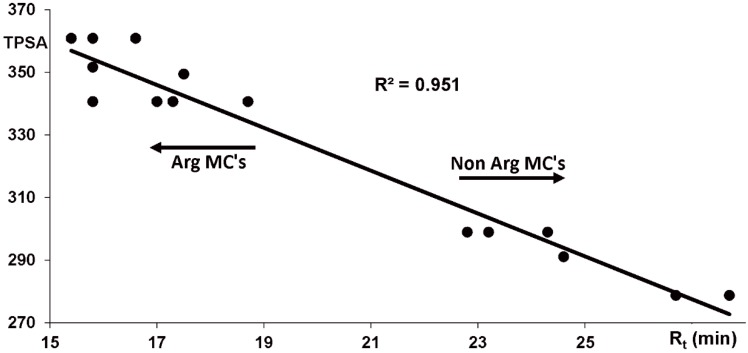
Effect of polar surface areas (PSA) to the retention times (R_t_, min) of *Phormidium* sp. LP904c microcystins calculated with a topological polar surface area (TPSA) method [30]. High correlation (R² = 0.951) shows that the proposed microcystin structures fit well to the measured retention times.

In addition to the mass spectral behavior, the chromatographic retention behavior was consistent with the structures of microcystins produced by *Phormidium* spp. DVL1003c and LP904c (Supplementary Figures S2 and S3). Polar surface areas obtained with the topological method from the microcystin 3D molecular structures correlated well (R^2^ = 0.951) with the measured retention times obtained from reversed phase chromatography (Figure 3 and Supplementary Figure S7). Large macrocyclic structures with many polar groups did not give the best polar surface areas with a topological method. Despite this, Topological Polar Surface Area (TPSA) values of microcystins, which are macrocyclic and polar groups containing molecules correlated well with the retention times. These results show that there is no discrepancy between the derived microcystin structures and their surface polarities.

Microcystin contents of *Phormidium* spp. LM603a (5–11b), LM603d (12–29d) (Figure 1C), and LS703b (1–2, Figure 1D) strains were very similar with the *Phormidium* spp. DVL1003c and LP904c (Supplementary Figure S8). However, recent chemical analysis indicates that microcystin is no longer produced by *Phormidium* sp. DVL1003c and we decided to choose strain LP904c for further analysis.

### 2.2. Microcystin and Homophenylalanine Biosynthetic Genes from Phormidium

**Figure 4:**
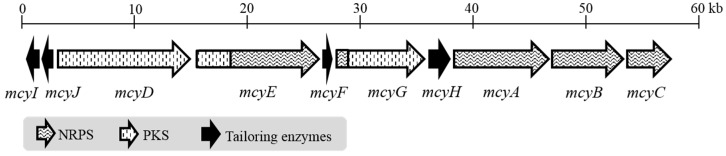
The 54.2 kb microcystin (*mcy*) gene cluster from *Phormidium* sp. LP904c. The respective sequences that will be transcribed to NRPS (nonribosomal peptide synthetase), PKS (polyketides synthase) and tailoring enzymes are indicated in the gene cluster.

A draft genome sequence from *Phormidium* sp. LP904c (7.7 Mb, 108 scaffolds, 129,707 N50) was obtained to identify the microcystin biosynthetic gene cluster (*mcy*) The complete 54.2 kb *mcy* gene cluster was identified through tBLASTn searches (Figure 4). The microcystin biosynthetic gene cluster from *Phormidium* sp. LP904c encodes ten genes organized in a bidirectional operon (Figure 4). There are three NRPS genes (*mcyA*, *mcyB* and *mcyC*), one PKS gene (*mcyD*), two hybrid NRPS-PKS genes (*mcyE* and *mcyG*), an aspartate racemase (*mcyF*), an ABC transporter (*mcyH*), a dehydrogenase (*mcyI*) and a methyltransferase (*mcyJ*) (Figure 4 and Supplementary Table S4). Most of the protein sequences from the enzymes of the microcystin biosynthetic pathway in *Phormidium* sp. LP904c are similar to sequences from *Planktothrix* spp. (Supplementary Table S4). The organization of *mcy* genes is identical to the *Planktothrix agardhii* CYA126 except for the positions of *mcyI* and *mcyJ* (Figure 4). Many of the microcystin variants produced by *Phormidium* sp. LP904c contain homo-amino acids (Figure 2 and Table 1). However, the *mcy* biosynthetic gene cluster from *Phormidium* sp. LP904c lacked obvious enzymes for the biosynthesis of homo-amino acids (Figure 4).

### 2.3. McyB1 and McyC from Phormidium sp. LP904c

The adenylation domains of McyB_1_ and McyC are responsible for the selection and activation of the amino acids in the positions X and Z of the microcystin, respectively. Sequence information based on microcystin biosynthetic genes from *Phormidium* sp. DVL1003c was obtained using PCR products based on specific primers prior to loss of microcystin biosynthesis by this strain [10]. These DNA sequences were translated to amino acid sequences and the predicted adenylation domain binding pockets of McyB_1_ and McyC from different cyanobacteria were obtained (Table 2 and Table 3). Both McyB_1_ and McyC adenylation domains binding pockets from *Phormidium* sp. LP904c and *Phormidium* sp. DVL1003c differ at the positions 236, 239 and 278 (in addition to position 331 in the McyB_1_ and exception in position 278 in McyC of DVL1003c) when compared to the same adenylation domains from other strains previously described in the literature (Table 2 and Table 3).

**Figure 5:**
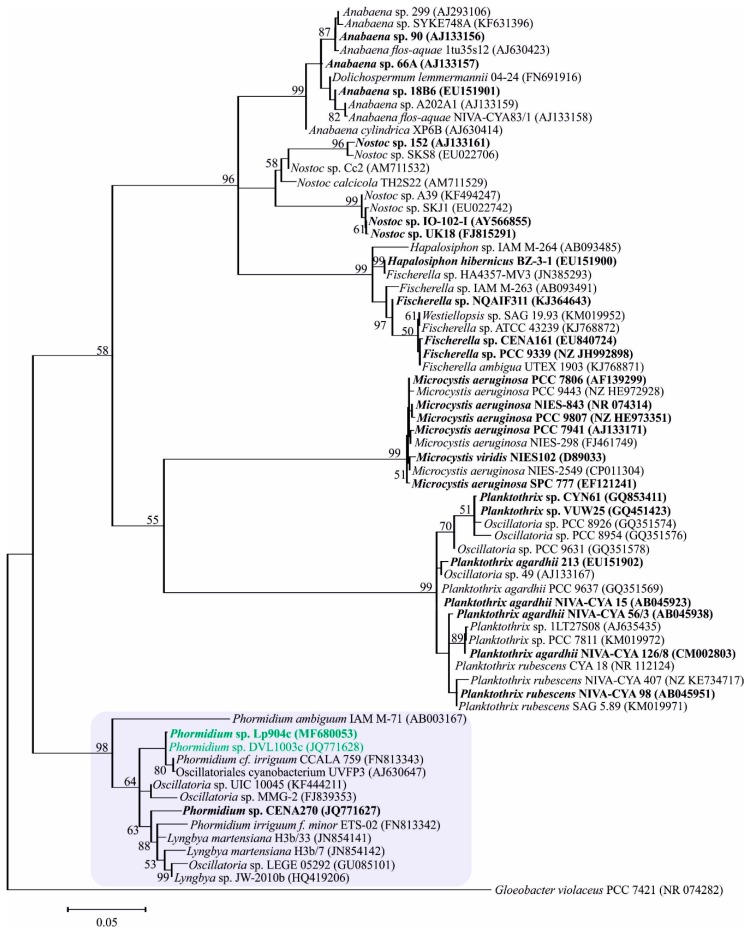
A phylogenetic tree of the 16S rRNA gene sequences constructed using maximum likelihood with 1000 bootstrap. Strains detected producing microcystin are highlighted in bold and the *Phormidium* spp. DVL1003c and LP904c studied here are highlighted in green. Planktonic *Anabaena* have recently been taxonomically assigned to the genus *Dolichospermum*.

Phylogenetic analysis based on the conserved sequences of 16S rRNA genes indicates the relationship of the studied strain to the other cyanobacteria sequences available in the National Center for Biotechnology Information (NCBI) GenBank database (Figure 5). *Phormidium* spp. DVL1003c and LP904c are grouped with other *Phormidium*, *Oscillatoria* and *Lyngbya* strains in the 16S rRNA gene phylogenetic tree (Figure 5). Other strains producing microcystin belonging to the order Nostocales, Oscillatoriales, Stigonematales and Chroococcales were included in the phylogenetic tree of the 16S rRNA gene and a broad distribution of these strains based on their relativeness to similar strains can be seen (Figure 5).

The concatenated phylogenetic tree based on microcystin biosynthetic enzymes McyD and McyE indicates the relationship of the *Phormidium* sp. LP904c sequences and other cyanobacteria producers of microcystins (Supplementary Figure S9). The studied strains are grouped with close related *Phormidium* and *Planktothrix* strains. However, recombination can be seen analyzing the phylogenetic history based on condensation and adenylation domains from the McyB_1_ and McyC (Supplementary Figure S10). Condensation domains sequences are divided into two big groups according to each enzyme: McyB_1_ and McyC (Supplementary Figure S10A). In contrast, the adenylation domain phylogenetic tree shows the recombination events occurred between the gene sequences (Supplementary Figure S10B). Adenylation domains from *Phormidium* spp. LP904c and DVL1003c McyB_1_ are grouped with domains from McyB_1_ and McyC from *Dolichospermum* and *Hapalosiphon* (Supplementary Figure S10B), while the adenylation domain from McyC in *Phormidium* sp. LP904c and DVL1003c is grouped with sequences from the McyB_1_ domains from *Planktothrix* spp.

## 3. Discussion

### 3.1. Benthic Cyanobacteria Producing Unusual Microcystins

Cyanobacteria are known for the production of toxins implicated in the toxicosis of animals and humans [2]. The hepatotoxin microcystin producing cyanobacteria have been detected mainly from planktonic strains present in freshwater environments distributed worldwide [2]. However, microcystins have been reported from benthic *Fischerella* and *Phormidium* strains [13,23,24,25,26,34,35]. *Phormidium* sp. LP904c was previously described to produce a high amount (441 ± 109 µgL^−1^) of microcystin and the main variant detected was microcystin-LR [24]. The present study shows the benthic *Phormidium* sp. LP904c producing altogether 16 microcystins. Different isolates from diverse genera of cyanobacteria have been related to the hepatotoxin production, such as *Anabaena*/*Dolichospermum* spp., *Nostoc* sp., *Plectonema* sp. and *Phormidium* sp. from Egypt [23], *Phormidium* sp. from USA, New Zealand and Brazil [13,24,26] and *Fischerella* sp. from Brazil and Australia [34,35,36]. Microcystin has been detected in benthic environmental samples from diverse places worldwide, including Switzerland [37], Spain [38,39], Australia [40], Antarctica [41] and the Arctic [42]. These wide distributions of toxic samples and the rise of the number of cyanobacterial isolates from diverse places in the world highlights the need to monitor these benthic communities for toxin production.

### 3.2. Microcystins Containing Homo-Amino Acids

The results show *Phormidium* sp. LP904c producing, in addition to the main variant microcystin-LR (55%), a variation of amino acids in position X and Z (Figure 2, Table 1). Leucine, arginine, phenylalanine, tryptophan, tyrosine, methionine and rare homo-amino acids Hph and Hty were detected (Figure 2, Table 1). Microcystins varying in different parts of its chemical structure were also detected, such as D-Asp^3^, L-MeSer^7^, Dha^7^ and DMAdda^5^ (Figure 2, Table 1). Microcystins are synthesized by nonribosomal peptide synthetases and polyketide synthases [5,9]. These nonribosomal pathways are known for the ability to incorporate proteinogenic and non-proteinogenic amino acids to the growing peptide chain. *Phormidium* sp. LP904c has been shown in this study to produce diverse variants of microcystins containing Hty and Hph at positions X and Z (Figure 2 and Table 1). Altogether, 31% of the microcystin variants produced contained homo-amino acids. The homo-amino acids are 14 mass units bigger than the proteinogenic amino acid due the presence of a methylene (CH_2_) group in the carbon side chain. *Phormidium* sp. CENA270 isolated from Brazil produces a range of [D-Leu^1^] microcystin variants, including Har at the position Z [13]. Hty has been found in diverse cyanobacterial compounds, such as aeruginosins, cyanopeptolins and microginins [9,43]. Hph was detected in pahayokolides [44] and microcystin [45]. Some microcystins, spumigins, lyngbyazothrins, tychonamides, schizotrin A, portoamides and anabaenopeptins contain Hph and/or Hty in their chemical structure [9,15,46,47,48]. HphA, HphB and HphCD were shown to be involved in the synthesis of Hph from L-Phe in *Nostoc punctiforme* PCC 73102 [14]. Interestingly, *Escherichia coli* containing the cyanobacterial *hph* genes could also convert L-Tyr to Hty [14]. *Phormidium* sp. LP904c has the *hphABCD* genes present in another part of the genome as part of the anabaenopeptin gene cluster [16]. The *mcy* biosynthetic gene cluster of *Phormidium* sp. LP904c did not encode enzymes for the biosynthesis of homo-amino acids and it is plausible that the McyB_1_ and McyC adenylation domains activate the homo-amino acids produced as part of the biosynthesis of anabaenopeptins.

### 3.3. Microcystin Gene Cluster from Phormidium

The microcystin gene cluster has been described from *Microcystis*, *Planktothrix*, *Dolichospermum*, *Nostoc* and *Fischerella* [5,6,7,8,49]. In this study, the microcystin biosynthetic gene cluster from *Phormidium* sp. LP904c is presented. The *Phormidium* microcystin biosynthetic enzymes have high similarity to *Planktothrix* amino acid sequences and it is as well mostly unidirectional, with exception of *mcyI* (dehydrogenase) and *mcyJ* (methyltransferase) (Figure 4 and Supplementary Table S4). However, the thioestherase McyT present in *Planktothrix agardhii* CYA 126 [6] is absent in the *Phormidium* microcystin gene cluster. The new *mcy* gene cluster sequence further expands the known genetic diversity of the microcystin family and will allow methods to detect the toxin based on gene sequences to be further refined.

The adenylation domains from the NRPSs McyB_1_ and McyC are responsible for the selection and activation of the amino acid to be incorporated in the positions X and Z of microcystin, respectively [5,6,7,8]. In the present study, the gene regions of *mcyB1* and *mcyC* from *Phormidium* sp. LP904c were analyzed to address their variation compared with previously described adenylation domains binding pockets. The adenylation domains have eight to ten amino acids responsible for the substrate specificity in the NRPS [31,50,51]. The binding pocket in the McyB1 and McyC adenylation domains from *Phormidium* sp. LP904c are unusual compared to other adenylation domains from other strains producing microcystins (Table 2 and Table 3). The McyC binding pocket is predicted to activate leucine, even though no microcystins with a leucine in the position Z were detected. The adenylation domains of McyA1, McyA2, McyB1 and McyC were found to be affected by recombination events which might have resulted in the high number of chemical variants in the positions 7, 1, 2 and 4 of the microcystin, respectively [10,11,13,52,53,54,55,56,57]. The adenylation domain sequences of McyB1 and McyC from *Phormidium* sp. LP904c might have gone through recombination events because their sequences group with sequences from different enzymes (Supplementary Figure S10). Adenylation domains from nostopeptolide [58] and cyanopeptolin [59] biosynthetic pathways have been shown to activate more than one amino acid. The synthesis of high numbers of microcystin variants could be attributed to the multispecificity of the adenylation domains, specificity-regulation and gatekeeping function of the condensation domains, tolerance of the condensation domains for recombination and point mutations after recombination events that change the specificity of the adenylation-condensation domains [60]. Further analysis based on biochemical assays of adenylation domains could improve the knowledge of amino acids incorporated by these systems.

### 3.4. Lack of Microcystin from Phormidium sp. DVL1003c

*Phormidium* sp. DVL1003c was first analyzed by our group for the synthesis of microcystin in 2005 and documented on the microcystin variants and *mcy* synthetase genes. In addition, Izaguirre and collaborators (2007) reported the synthesis of microcystin-LR by this strain [24]. Surprisingly, the culture maintained in the University of Helsinki Culture Collection is no longer able to produce microcystins. Other *Phormidium* strains which have been cultivated under identical conditions still produce almost the same microcystins variants as DVL1003c was able to produce (Supplementary Figure S8). Thus, this study presents one example of a cyanobacterial strain which loses the ability to synthesize a natural product under laboratorial conditions.

### 3.5. Diversity of Benthic Phormidium Strains

*Phormidium* spp. isolated from Egypt, USA, Brazil and Spain have been reported to produce microcystins [23,24,25]. In the phylogenetic tree constructed with 16S rRNA gene sequences, microcystin-producing strains of *Phormidium* are grouped together with other Oscillatoriales such as *Oscillatoria* and *Lyngbya* (Figure 5). The low taxonomic agreement among these strains opens the possibilities for discovery of new benthic cyanobacteria yet to be assigned to some genera and that can be a potential microcystin producer.

## 4. Materials and Methods

### 4.1. Cultivation of Phormidium Strains

The *Phormidium* strains were isolated from Riverside County, California, USA: DVL1003c was isolated from a freshwater reservoir, strains LM603a (5–11b) and LM603d (12–29d) were isolated from Lake Mathews, strain LP904c (3–7b) from Lake Perris and strain LS703b (1–2) from Lake Skinner [24]. The cultures were grown in 50 mL of Z8 medium at 22 °C under constant light.

### 4.2. Chemical Analysis

Freeze dried cyanobacterial biomass was extracted with methanol as previously described [13]. Methanol extracts were analyzed with low resolution HPLC-ESI-ITMS (Agilent 1100 Series LC/MSD Ion Trap XCT Plus, Agilent Technologies, Palo Alto, CA, USA) to obtain the chromatographic data and product ion spectra of protonated microcystins. A 10 μL sample was injected into a Zorbax C8 column (4.6 × 150 mm, 5 μm, Agilent technologies, Palo Alto, CA, USA) which was eluted for 7.5 min isocratically with 5% isopropanol (+0.05% trifluoracetic acid (TFA)) (solvent B) in 0.05% TFA then to 16% of B in 2.5 min, to 46% of B in 40 min, to 100% of B in 10 min and finally isocratically for 5 min at 40 °C with a flow rate of 0.6 mL min^−1^ so that the total length of the elution program was 65 min. Mass spectral data was accumulated in Ultra Scan positive electrospray ionization mode (26,000 *m*/*z* s^−1^) at scan range of *m*/*z* 100–1200 and by averaging three spectra.

High resolution UPLC-QTOF (Acquity I-Class UPLC-Synapt G2-Si HDMS, Waters Corp., Milford, MA, USA) analyses of microcystins were performed from the methanol extract of *Phormidium* sp. LP904c (3–7b). One μL sample was injected to Cortecst UPLC^®^ C18+ column (2.1 × 50 mm, 1.6 μm, Waters) which was eluted at 40 °C with a flow rate of 0.3 mL min^−1^ from 20% acetonitrile (+0.1% HCOOH) (solvent B) in 0.1% HCOOH to 95% of B in 5 min, was kept there for 2 min, then back to 20% of B in 0.5 min and finally kept there for 2.5 min before next run. QTOF was calibrated with sodium formate giving a calibrated mass range of *m*/*z* 91.036–1178.651. Leucine enkephalin was used at 10 s intervals as a lock mass reference compound. Mass spectral data was accumulated in positive electrospray ionization Resolution Mode at scan range of *m*/*z* 50–1200.

The chemical characterization of certain microcystin variants was analyzed based on HPLC-ESI-ITMS and UPLC-QTOF results. Microcystin structures derived from mass spectral data was matched with chromatographic retention behavior. Polar surface areas of microcystins were calculated with a topological method [30], which gave TPSA (topological polar surface area) values for the microcystins.

### 4.3. DNA Extraction, PCR and Genome Sequencing and Assembly

The genomic DNA extraction from strain *Phormidium* sp. DVL1003c was obtained as described in [10]. Fragments of the 16S rRNA (JQ771628.1) and *mcyB1CDE* genes were obtained by PCR and sequenced as described in [10]. Alternatively, the genomic DNA extraction from strain *Phormidium* sp. LP904c was obtained as described in [15,61]. An isolated DNA sample from *Phormidium* sp. LP904c was checked using a NanoDrop 1000 spectrophotometer (Thermo Scientific) to measure the concentration and an Agilent TapeStation (Agilent Technologies) to assess the quality. High-molecular DNA was subjected to library (Illumina TruSeq^®^ PCR Free 350bp, Illumina, San Diego, CA, USA) construction and sequenced by Illumina HiSeq2500 platform (Illumina, San Diego, CA, USA) with a paired ends 100 cycles run. The genome data (1 Gb) of *Phormidium* sp. LP904c was firstly checked by Spades (version 3.7.1) [62] for read correction and removal of erroneous ones, and then assembled using Newbler (version 3.0) [63]. Non-cyanobacterial sequences from contaminants were identified by Kraken 1.0 [64] and removed using a custom script. The accession number of complete sequences of the microcystin gene cluster from *Phormidium* sp. LP904c is MK870090 and the *mcyB1CDE* genes from *Phormidium* sp. DVL1003c are MK924153-MK924156.

### 4.4. Bioinformatics Analysis

These sequences obtained from *mcyB1C* were translated using BioEdit [65]. The predictions of the binding pockets of McyC and McyB1 adenylation domains was performed using the bioinformatics tool PKS/NRPS analysis [66], alignment of the sequences using BioEdit [47] and NRPSpredictor2 [32,33]. The phylogenetic tree based on 16S rRNA gene sequences was constructed in Molecular Evolutionary Genetic Analysis (MEGA) 5 [67] using the maximum likelihood method (K2+G+I model) and 1000 bootstrap replicates. A concatenated phylogenetic tree constructed using McyD and McyE sequences was obtained using the neighbor-joining method (Poisson model + G) and 1000 bootstrap replications. The same parameters were used to construct the phylogenetic tree using adenylation and condensation domains from McyB1 and McyC.

## 5. Conclusions

Benthic cyanobacteria belonging to *Phormidium* genus isolated from freshwater environments were shown to produce microcystin containing homo-amino acids. This finding increases the knowledge of microcystin variants produced by *Phormidium*. Furthermore, we describe the microcystin biosynthetic genes from *Phormidium* sp. LP904c, which resembles the ones found in *Planktothrix* strains. Interestingly, *Phormidium* sp. DVL1003c ceased the synthesis of microcystins during the laboratorial maintenance in our laboratory. Further work is necessary to unveil why two strains kept under the same cultivation conditions had different fates.

## Figures and Tables

**Table 1:** Protonated ion masses of microcystin (MC) variants from *Phormidium* sp. LP904c and their relative amounts (%) identified from either *Phormidium* sp. LP904c or DVL1003c by HPLC-ESI-ITMS. Peak areas of the extracted ion chromatograms of the protonated microcystins were used in relative amount calculations. Hty = homotyrosine, Hph = homophenylalanine, [M + H]^+^ (*m*/*z*) = mass per charge value of protonated microcystins, R_t_ (min) = retention time in minutes, RA = relative amount, Aa = amino acid. Additional information presented in Figure S1.

No.	Microcystin	Aa in Position		Rt	[M + H]^+^ (*m*/*z*)		Error	RA (%)	
		X	Z	(min)	Calculated	Measured	(ppm)	LP904c	DVL1003c
1	MC-YR	**Y**	**R**	15.4	1045.5353	1045.5329	–2.35	<1	<1
2	MC-HtyR	**Hty**	**R**	15.8	1059.5510	1059.5510	–0.01	4	4
3	MC-MR	**M**	**R**	15.8	1013.5125	1013.5148	2.26	1	1
4	[DMAdda^5^]MC-LR	**L**	**R**	15.8	981.5404	981.5404	–0.04	<1	<1
5	[L-MeSer^7^]MC-LR	**L**	**R**	16.6	1013.5666	1013.5649	–1.73	<1	<1
6	[D-Asp^3^]MC-LR	**L**	**R**	17	981.5404	981.5404	–0.04	<1	<1
7	MC-LR	**L**	**R**	17	995.5560	995.5566	0.51	55	49
8	MC-FR	**F**	**R**	17.3	1029.5404	1029.5401	–0.33	9	7
9	[Dha^7^]MC-LR	**L**	**R**	17.5	981.5404	981.5404	–0.04	<1	<1
10	MC-HphR	**Hph**	**R**	18.7	1043.5560	1043.5561	0.01	17	14
11	MC-LY	**L**	**Y**	22.8	1002.5183	1002.5178	–0.51	1	2
12	MC-LHty	**L**	**Hty**	23.2	1016.5339	1016.5347	0.73	5	12
13	MC-HphHty	**Hph**	**Hty**	24.3	1064.5339	1064.5337	–0.24	3	8
14	MC-LW	**L**	**W**	24.6	1025.5342	1025.5343	0.00	<1	<1
15	MC-LHph	**L**	**Hph**	26.7	1000.5390	1000.5369	–2.15	<1	1
16	MC-HphHph	**Hph**	**Hph**	27.7	1048.5390	1048.5403	1.20	<1	<1

**Table 2:** Specificity codes inferred from the protein sequence of first adenylation domain of McyB_1_ according to [31] or NRPSpredictor2 [32,33]. Conserved amino acids are highlighted in light blue and variable amino acids are highlighted in dark green. Homo-amino acids are highlighted in red.

Organism	Strain	McyB1										P *	%	Aa ^#^
		235	236	239	278	299	301	322	330	331	517			
*Phormidium* sp.	LP904c	D	I	C	V	F	G	L	V	H	K	Ser-Thr	60	Leu, Hph, Phe, Hty, Tyr, Arg
*Phormidium* sp.	DVL1003c	D	I	C	V	F	G	L	V	H	K	Ser-Thr	60	Leu, Hph, Phe, Hty, Tyr, Arg
*Planktothrix agardhii*	CYA 126/8	D	A	L	F	F	G	L	V	D	K	Leu	100	Arg, Leu
*Planktothrix agardhii*	213	D	A	L	F	F	G	L	V	D	K	Leu	100	Arg, Leu
*Planktothrix agardhii*	NIVA-CYA56/3	D	A	L	F	F	G	L	V	D	K	Leu	100	Leu, Arg, Tyr
*Planktothrix prolifica*	NIVA-CYA 98	D	A	L	F	F	G	L	V	D	K	Leu	100	Leu, Arg
*Planktothrix rubescens*	NIVA-CYA 407	D	A	L	L	F	G	L	V	D	K	Leu	90	Leu, Arg, Hty
*Dolichospermum* sp. ^∞^	90	D	V	W	F	F	G	L	V	D	K	Ser	80	Leu, Arg, Hil
*Dolichospermum flos-aquae* ^∞^	18B6	D	V	W	S	F	G	L	V	D	K	Ser	80	Arg, X
*Dolichospermum lemmermannii* ^∞^	66 A	D	V	W	S	F	G	L	V	Y	K	Ser	70	Hty, X, Hph, Leu, Tyr, Phe
*Nostoc* sp.	152	D	A	L	F	F	G	L	I	Y	K	Leu	80	Leu, Hil, X, Val
*Nostoc* sp.	IO-102-I	D	I	K	N	F	G	A	I	V	K	Gln	50	Leu, X, Phe, Hil, Tyr
*Fischerella* sp.	PCC9339	D	V	L	I	F	G	L	I	Y	K	Pro	70	Leu
*Hapalosiphon hibernicus*	BZ-3-1	D	V	W	F	F	G	L	V	D	K	Ser	80	Leu, Arg
*Microcystis aeruginosa*	PCC 7806	D	A	W	F	L	G	N	V	V	K	Leu	100	Val
*Microcystis aeruginosa*	FCY-28	D	G	W	T	I	G	A	V	E	K	Arg	90	Leu
*Microcystis aeruginosa*	FCY-26	D	G	W	T	I	G	A	V	E	K	Arg	90	Ni
*Microcystis aeruginosa*	UV027	D	V	W	T	I	G	A	V	E	K	Arg	100	Arg
*Microcystis aeruginosa*	K139	D	A	W	F	L	G	N	V	V	K	Leu	100	Leu
*Microcystis aeruginosa*	DIANCHI905	D	A	W	F	L	G	N	V	V	K	Leu	100	Ni
*Microcystis aeruginosa*	PCC 9807	D	A	W	F	L	G	N	V	V	K	Leu	100	Ni
*Microcystis aeruginosa*	PCC 7941	D	A	W	F	L	G	N	V	V	K	Leu	100	Ni
*Microcystis aeruginosa*	PCC 9443	D	G	W	T	I	G	A	V	E	K	Arg	90	Ni
*Microcystis aeruginosa*	NIES-843	D	G	W	T	I	G	A	V	E	K	Arg	90	Arg, Leu, Tyr
*Microcystis aeruginosa*	SPC777	D	G	W	T	I	G	A	V	E	K	Arg	90	Arg
*Microcystis viridis*	NIES 102	D	G	W	T	I	G	A	V	E	K	Arg	90	Hil, Leu, Arg, Tyr, Trp, Phe, Hty, X

P * prediction by NRPSpredictor2; Aa ^#^ amino acid detected by LC-MS (Supplementary Table S5); X. MC contains an unknown amino acid or the overall amino acid content is not known; Ni. No information. ^∞^ Planktonic *Anabaena* have recently been taxonomically assigned to the genus *Dolichospermum*.

**Table 3:** Specificity codes inferred from the protein sequence of the adenylation domain of McyC according to [31] or NRPS predictor2 [32,33]. Conserved amino acids are highlighted in light blue and variable amino acids are highlighted in dark green. Homo-amino acids are highlighted in red.

Organism	Strain	McyC										P *	%	Aa ^#^
		235	236	239	278	299	301	322	330	331	517			
*Phormidium* sp.	LP904c	D	A	L	F	F	G	L	V	D	K	Leu	100	Arg, Hty, Hph, Trp
*Phormidium* sp.	DVL1003c	D	A	L	C	F	G	L	V	D	K	Leu	100	Arg, Hty, Hph, Trp
*Planktothrix agardhii*	CYA 126/8	D	P	W	G	F	G	L	V	D	K	Gln	70	Arg
*Planktothrix agardhii*	213	D	P	W	C	F	G	L	V	D	K	Gln	70	Arg
*Planktothrix agardhii*	NIVA-CYA 56/3	D	P	W	G	F	G	L	V	D	K	Gln	70	Arg
*Planktothrix prolifica*	NIVA-CYA 98	D	P	W	G	F	G	L	V	D	K	Gln	70	Arg
*Planktothrix rubescens*	NIVA-CYA 407	D	P	W	G	F	G	L	V	D	K	Gln	70	Arg
*Dolichospermum* sp. ^∞^	90	D	V	W	C	F	G	L	V	D	K	Ser	80	Arg
*Dolichospermum flos-aquae* ^∞^	18B6	D	V	W	S	F	G	L	V	D	K	Ser	80	Arg
*Dolichospermum lemmermannii* ^∞^	66 A	D	V	W	S	F	G	L	V	D	K	Ser	80	Arg
*Nostoc* sp.	152	D	V	W	N	F	G	F	I	D	K	Gln	70	Arg, Har
*Nostoc* sp.	IO-102-I	D	V	W	N	F	G	F	V	D	K	Glu	70	Arg
*Fischerella* sp.	PCC9339	D	V	W	F	F	G	L	V	D	-	Ser	70	Arg
*Hapalosiphon hibernicus*	BZ-3-1	D	V	W	F	F	G	L	V	D	K	Ser	80	Ala, Leu, Val
*Microcystis aeruginosa*	PCC 7806	D	V	W	T	I	G	A	V	D	K	Arg	100	Arg
*Microcystis aeruginosa*	FCY-28	D	V	W	T	I	G	A	V	D	K	Arg	100	Ni
*Microcystis aeruginosa*	FCY-26	D	V	W	T	I	G	A	V	D	K	Arg	100	Ni
*Microcystis aeruginosa*	UV027	D	V	W	T	I	G	A	V	D	K	Arg	100	Arg
*Microcystis aeruginosa*	K139	D	V	W	T	I	G	A	V	E	K	Arg	100	Arg
*Microcystis aeruginosa*	DIANCHI905	D	V	W	T	I	G	A	V	D	K	Arg	100	Ni
*Microcystis aeruginosa*	PCC 9807	D	V	W	T	I	G	I	V	D	K	Arg	90	Ni
*Microcystis aeruginosa*	PCC 7941	D	V	W	T	I	G	A	V	D	K	Arg	100	Ni
*Microcystis aeruginosa*	PCC 9443	D	V	W	T	I	G	I	V	D	K	Arg	90	Ni
*Microcystis aeruginosa*	NIES-843	D	V	W	T	I	G	A	V	D	K	Arg	100	Arg
*Microcystis aeruginosa*	SPC777	D	V	W	T	I	G	A	V	D	K	Arg	100	Arg
*Microcystis viridis*	NIES 102	D	V	W	T	I	G	A	V	D	K	Arg	100	Arg

P * prediction by NRPSpredictor2; Aa ^#^ amino acid detected by LC-MS (Supplementary Table S5); X. MC contains an unknown amino acid or the overall amino acid content is not known; Ni. No information. ^∞^ Planktonic *Anabaena* have recently been taxonomically assigned to the genus *Dolichospermum*.

## Supplementary Materials

The following are available online at https://www.mdpi.com/1660-3397/17/5/271/s1: Figure S1: Relative amounts (%) of microcystin variants in *Phormidium* sp. LP904c (yellow) and DVL1003c (green).; Figure S2: Ultraviolet (UV), total ion current (TICC) and extracted ion (EIC) chromatograms obtained with HPLC-ITMS of protonated microcystins found from *Phormidium* sp. LP904c.; Figure S3: Ultraviolet (UV), total ion current (TICC) and extracted ion (EIC) chromatograms obtained with HPLC-ITMS of protonated microcystins found from *Phormidium* sp. DVL1003c.; Figure S4: Product ion spectra of protonated microcystins from *Phormidium* sp. LP904c obtained with HPLC-ITMS.; Figure S5: Coding for the product ions generated from the protonated and sodiated microcystins.; Figure S6: Product ion spectra of sodiated non Arg microcystins from *Phormidium* sp. LP904c obtained with HPLC-ITMS. Mass/charge values of protonated microcystins and their retention times (min) are marked to the spectra.; Figure S7: Effect of polar surface areas (PSA) to the retention times (R_t_, min) of *Phormidium* sp. DVL1003c microcystins calculated with a topological polar surface area (TPSA) method (Ertl et al., 2000).; Figure S8: Microcystin variants produced by studied *Phormidium* strains and relative amount produced.; Figure S9: Concatenated phylogenetic tree of the McyD and McyE amino acid sequences constructed using neighbor-joining with 1000 bootstrap replications.; Figure S10: Phylogenetic tree constructed using condensation (A) and adenylation (B) domains from McyB1 (blue) and McyC (pink) amino acid sequences.; Table S1: Assignments, ion masses (*m*/*z*) and intensities (%) of the protonated non Arg microcystins of the most important product ions.; Table S2: Assignments, ion masses (*m*/*z*) and intensities (%) of the sodiated non Arg microcystins of the most important product ions.; Table S3: Microcystin variants, retention times (R_t_), relative amounts (RA) and small diagnostic ions (from protonated MCs) with corresponding amino acids from *Phormidium* sp. LP904c by HPLC-ITMS and UPLC-QTOF. RAs were calculated from the sum of the peak areas of the extracted ion chromatograms of different ion species (H, Na, K and 2H) of microcystins.; Table S4: Sequence similarity of the microcystin gene cluster from *Phormidium* sp. LP904c obtained by BLASTp.
