# Supplementary material for: The Biosynthesis of Rare Homo-Amino Acid Containing Variants of Microcystin by a Benthic Cyanobacterium
Source: Mar Drugs. 2019 May 7;17(5):271. doi: 10.3390/md17050271 (PMC6562525; doi:10.3390/md17050271)
Supplement: Supplementary file 1 [file marinedrugs-17-00271-s001.pdf]

Supplementary material

The biosynthesis of rare homo-amino acid containing variants of microcystin by a benthic cyanobacterium

Tânia Keiko Shishido[1,2], Jouni Jokela[1], Anu Humisto[1], Hao Wang[1,3], Suvi Suurnäkki[1,4], Matti Wahlsten[1], Danillo O. Alvarenga[1], Kaarina Sivonen[1], David P. Fewer[1*]

Department of Microbiology, University of Helsinki, Viikinkaari 9, FI-0014, Helsinki, Finland; tania.shishido@helsinki.fi (T.K.S.); jouni.jokela@helsinki.fi (J.J.); anu.humisto@helsinki.fi (A.H.); matti.wahlsten@helsinki.fi (M.W.); danillo.oliveiradealvarenga@helsinki.fi (D.O.A.); kaarina.sivonen@helsinki.fi (K.S.)

[2] Institute of Biotechnology, Helsinki Institute of Life Science, University of Helsinki, Viikinkaari 5D, FI-0014, Helsinki, Finland

[3] Department of Biological and Environmental Science, University of Jyväskylä, FI-40014, Jyväskylä, Finland

[*] Correspondence: david.fewer@helsinki.fi; Phone: +358 9 19159270.

Figures

Figure S1. Relative amounts (%) of microcystin variants in *Phormidium* sp. LP904c (yellow) and DVL1003c (green). Peak areas of the extracted ion chromatograms of the protonated microcystins was used in calculations.

Figure S2. Ultraviolet (UV), total ion current (TICC) and extracted ion (EIC) chromatograms obtained with HPLC-ITMS of protonated microcystins found from *Phormidium* sp. LP904c. Mass/charge values of protonated microcystins are marked to the EIC traces.

Figure S3. Ultraviolet (UV), total ion current (TICC) and extracted ion (EIC) chromatograms obtained with HPLC-ITMS of protonated microcystins found from *Phormidium* sp. DVL1003c. Mass/charge values of protonated microcystins are marked to the EIC traces.

Figure S4. Product ion spectra of protonated microcystins from *Phormidium* sp. LP904c obtained with HPLC-ITMS. Mass/charge values of protonated microcystins and their retention times (min) are marked to the spectra.

Figure S5. Coding for the product ions generated from the protonated and sodiated microcystins. Variable methyl groups 1-3 are marked with a gray shade.

Figure S6. Product ion spectra of sodiated non Arg microcystins from *Phormidium* sp. LP904c obtained with HPLC-ITMS. Mass/charge values of protonated microcystins and their retention times (min) are marked to the spectra.

Figure S7. Effect of polar surface areas (PSA) to the retention times (R_t, min) of *Phormidium* sp. DVL1003c microcystins calculated with a topological polar surface area (TPSA) method (Ertl et al., 2000). High correlation (R^2 = 0,937) shows that the proposed microcystin structures fit well to the measured retention times.

Figure S8. Microcystin variants produced by studied *Phormidium* strains and relative amount produced.

Figure S9. Concatenated phylogenetic tree of the McyD and McyE sequences constructed using neighbor-joining with 1000 bootstrap replications. *Phormidium* sp. LP904c is indicated in bold.

Figure S10. Phylogenetic tree constructed using condensation (A) and adenylation (B) domains from McyB1 (blue) and McyC (pink) amino acid sequences. Neighbor-joining method with 1000 bootstrap replications. *Phormidium* sp. LP904c is indicated in bold.

Tables

Table S1. Assignments, ion masses (*m*/*z*) and intensities (%) of the protonated non Arg microcystins of the most important product ions. Red No = Ion structure confirmed by Diehnelt et al. 2006 with high resolution fourier transform ion cyclotron resonance mass spectrometer. CI = Code for ion structure presented in Figure S5.

Table S2. Assignments, ion masses (*m*/*z*) and intensities (%) of the sodiated non Arg microcystins of the most important product ions. CI = Code for ion structure presented in Figure S5.

Table S3. Microcystin variants, retention times (R_t), relative amounts (RA) and small diagnostic ions (from protonated MC's) with corresponding amino acids from *Phormidium* sp. LP904c by HPLC-ITMS and UPLC-QTOF. RA's were calculated from the sum of the peak areas of the extracted ion chromatograms of different ion species (H, Na, K and 2H) of microcystins. Hty = homotyrosine, Hph = homophenylalanine. Diagnostic ions of MC's No 14-16 were analysed from sodiated MC's and additionally MC No 15 ions masses were corrected with the mass of sodiated MC No 15 exact mass.

Table S4. Sequence similarity of the microcystin gene cluster from *Phormidium* sp. LP904c obtained by BLASTp.

Table S5. Microcystins variants detected in the strains from table 2 and 3. The homoamino acids detected are highlighted.

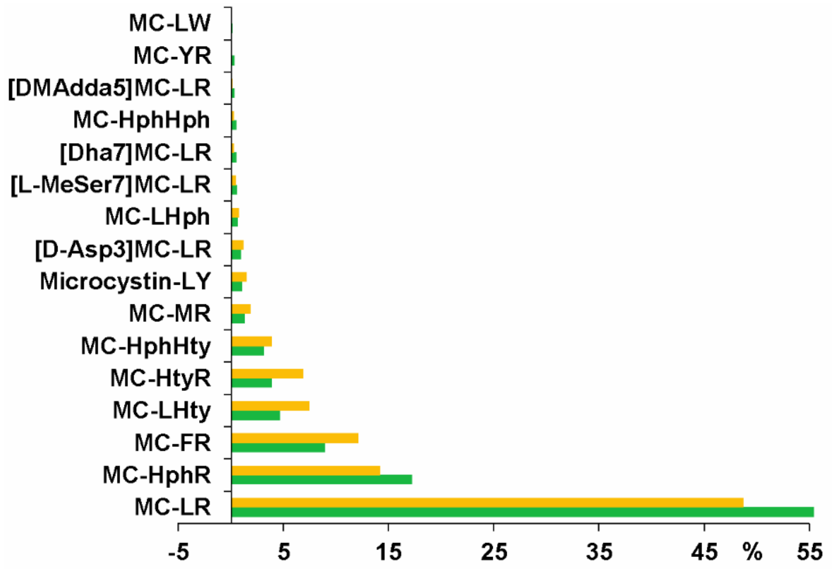

Figure S1. Relative amounts (%) of microcystin variants in *Phormidium* sp. LP904c (yellow) and DVL1003c (green). Peak areas of the extracted ion chromatograms of the protonated microcystins was used in calculations.

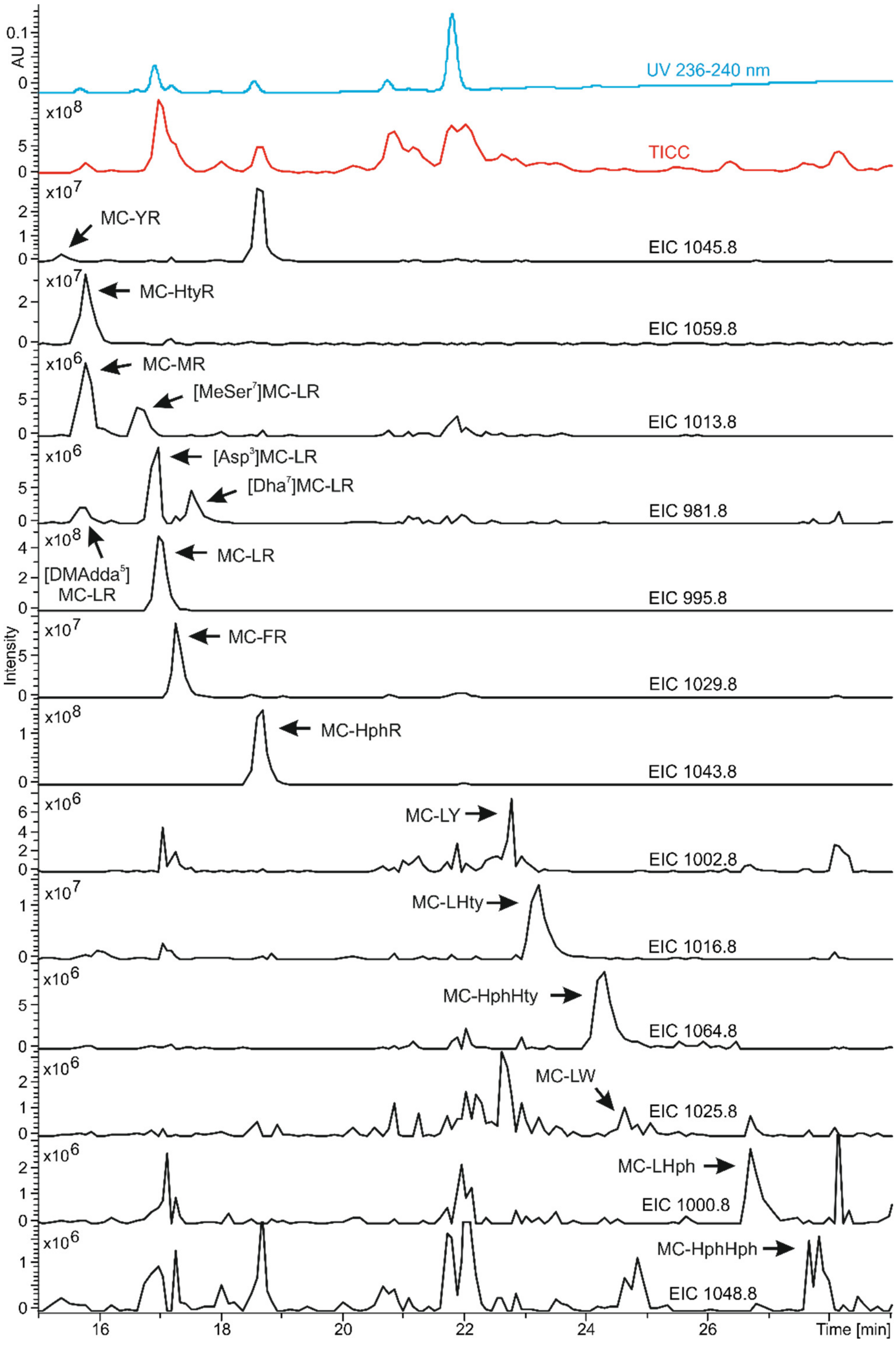

Figure S2. Ultraviolet (UV), total ion current (TICC) and extracted ion (EIC) chromatograms obtained with HPLC-ITMS of protonated microcystins found from *Phormidium* sp. LP904c. Mass/charge values of protonated microcystins are marked to the EIC traces.

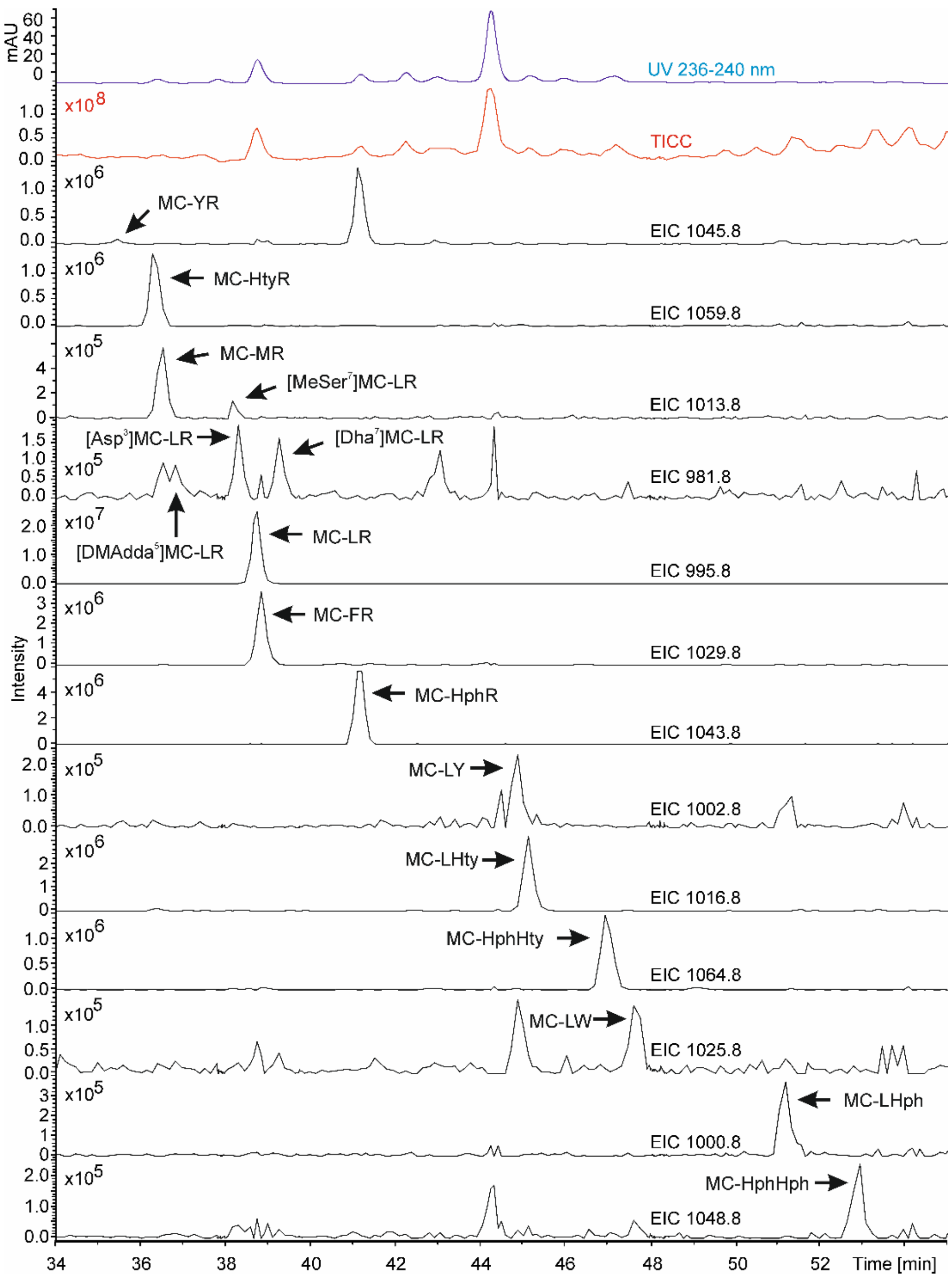

Figure S3. Ultraviolet (UV), total ion current (TICC) and extracted ion (EIC) chromatograms obtained with HPLC-ITMS of protonated microcystins found from *Phormidium* sp. DVL1003c. Mass/charge values of protonated microcystins are marked to the EIC traces.

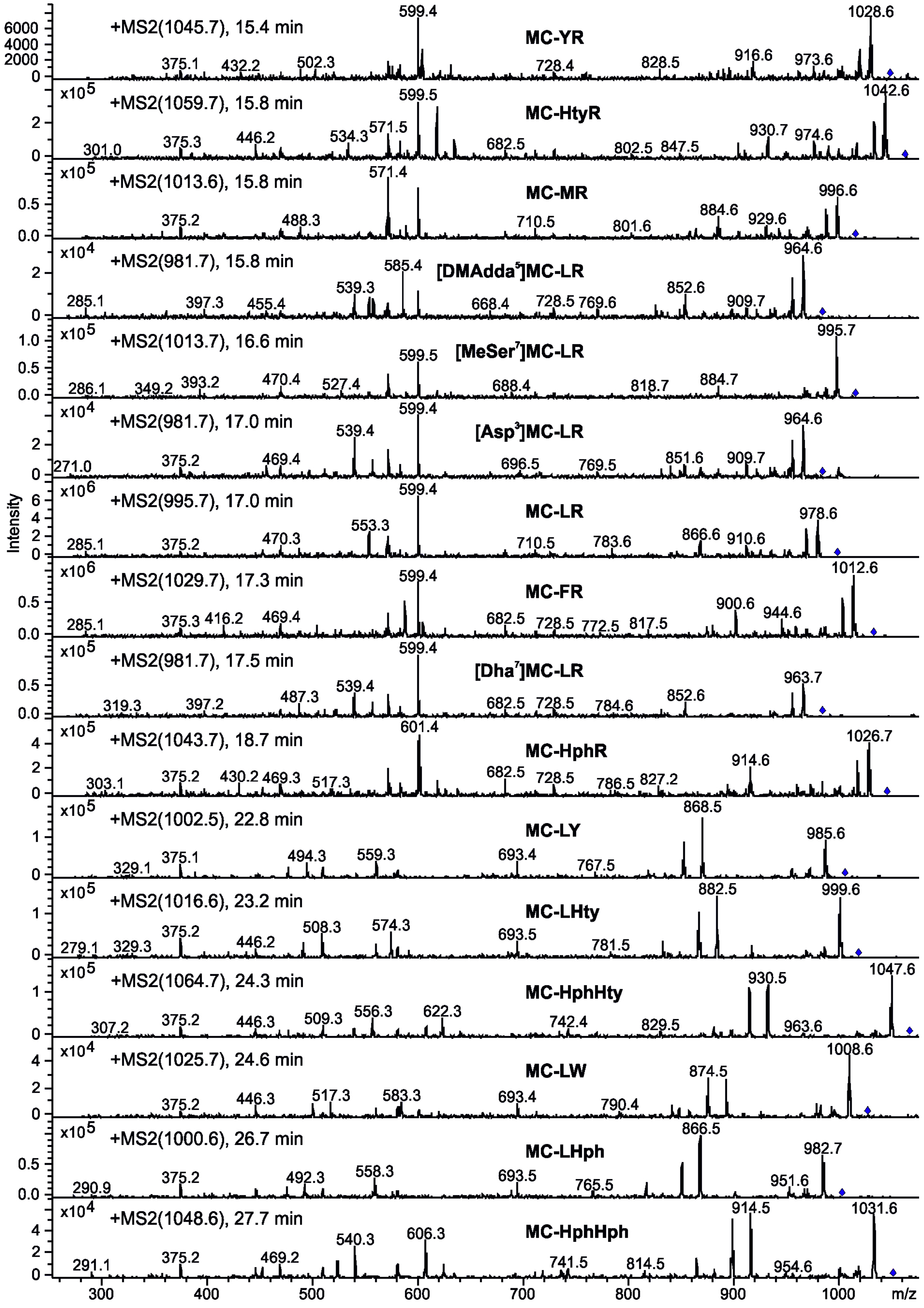

Figure S4. Product ion spectra of protonated microcystins from *Phormidium* sp. LP904c obtained with HPLC-ITMS. Mass/charge values of protonated microcystins and their retention times (min) are marked to the spectra.

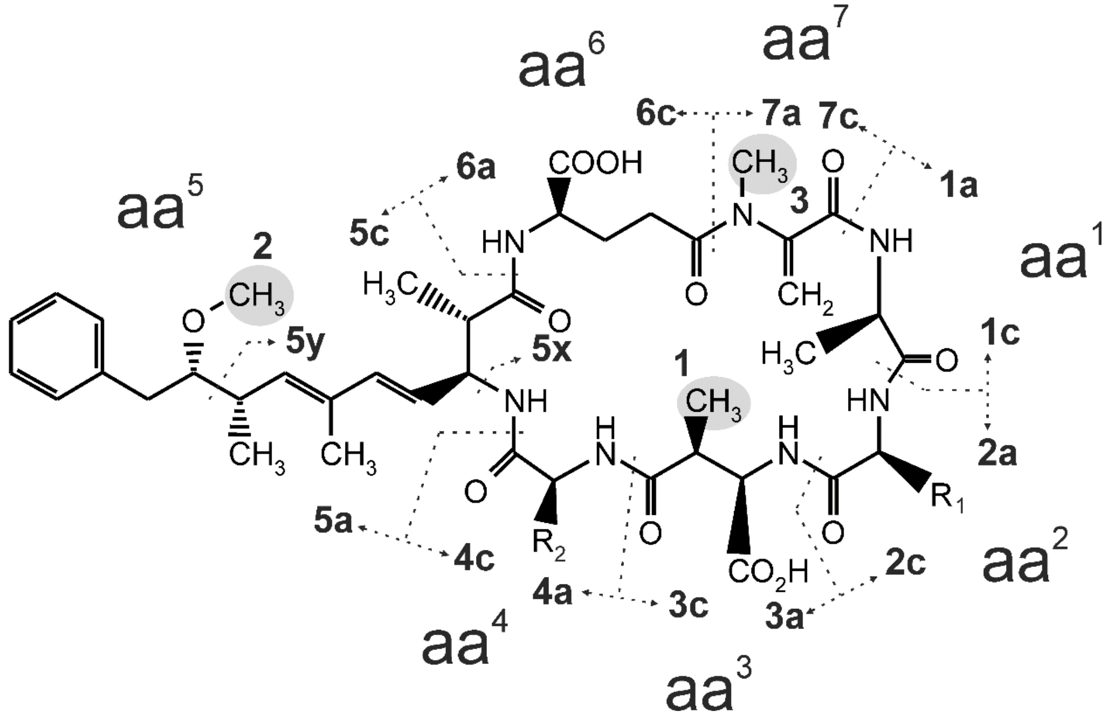

Figure S5. Coding for the product ions generated from the protonated and sodiated microcystins. Variable methyl groups 1-3 are marked with a gray shade.

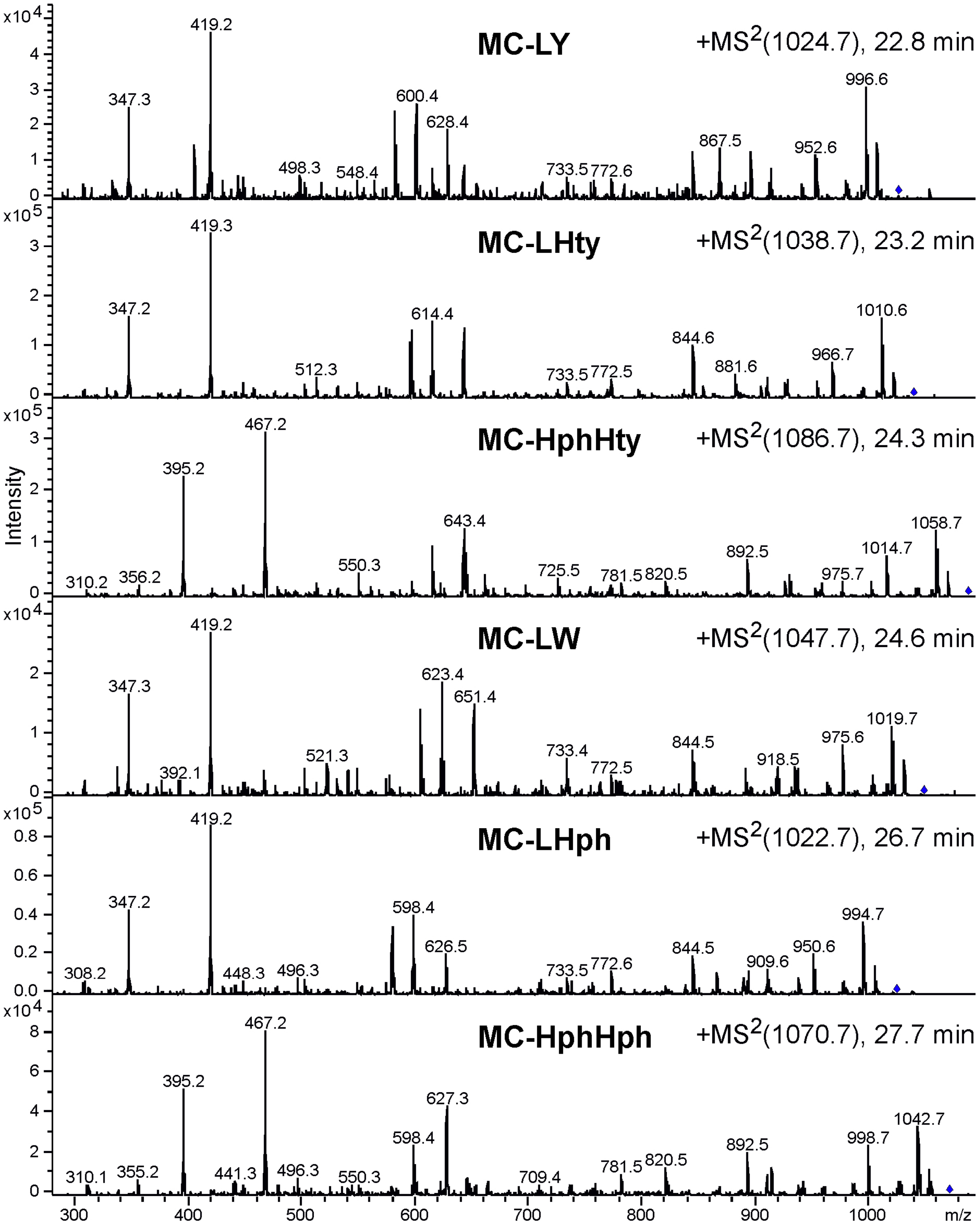

Figure S6. Product ion spectra of sodiated non Arg microcystins from *Phormidium* sp. LP904c obtained with HPLC-ITMS. Mass/charge values of protonated microcystins and their retention times (min) are marked to the spectra.

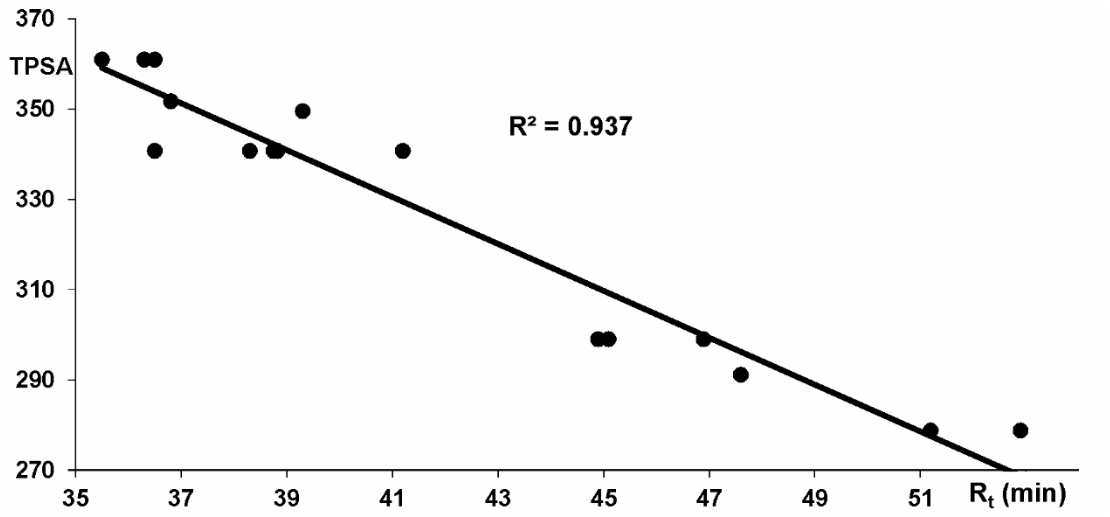

Figure S7. Effect of polar surface areas (PSA) to the retention times (R_t, min) of *Phormidium* sp. DVL1003c microcystins calculated with a topological polar surface area (TPSA) method (Ertl et al., 2000). High correlation (R^2 = 0,937) shows that the proposed microcystin structures fit well to the measured retention times.

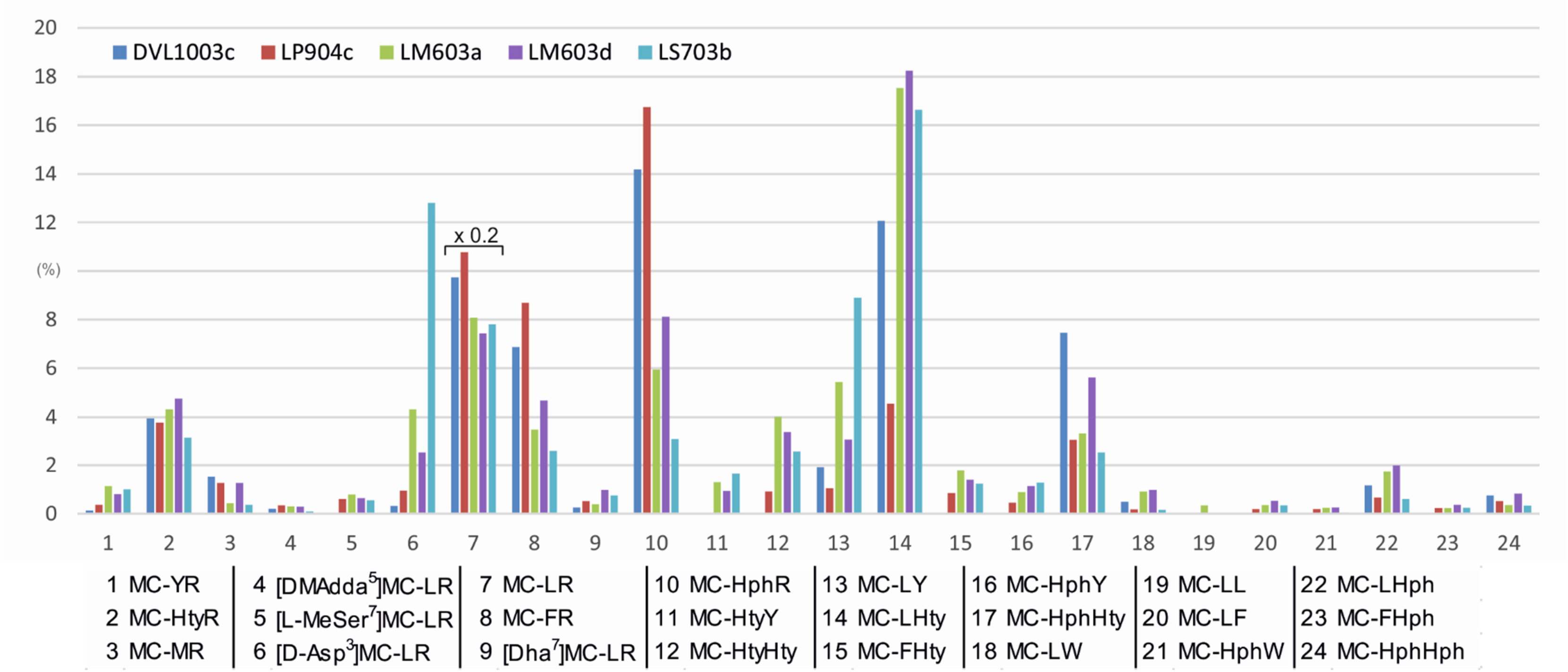

Figure S8. Microcystin variants produced by studied *Phormidium* strains and relative amount produced.

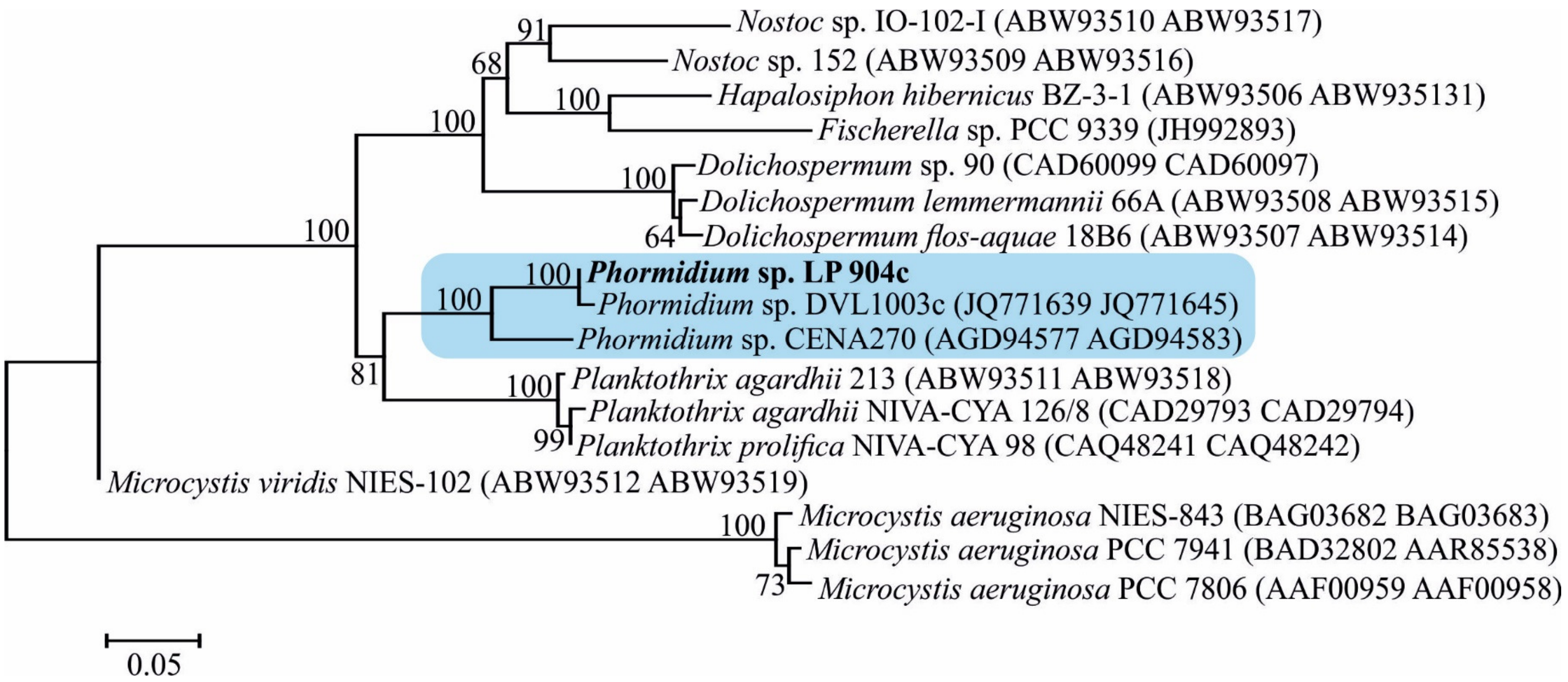

Figure S9. Concatenated phylogenetic tree of the McyD and McyE amino acid sequences constructed using neighbor-joining with 1000 bootstrap replications. *Phormidium* sp. LP904c is indicated in bold.

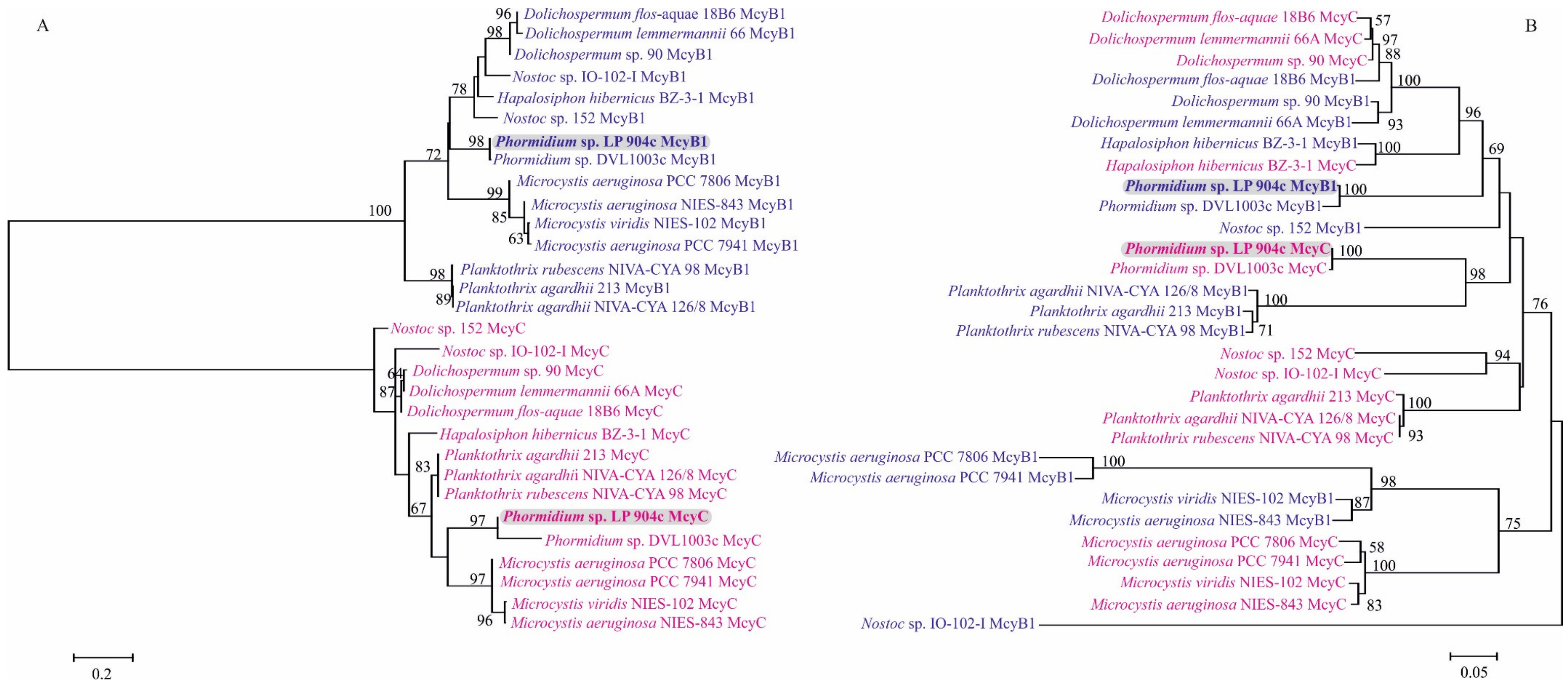

Figure S10. Phylogenetic tree constructed using condensation (A) and adenylation (B) domains from McyB1 (blue) and McyC (pink) amino acid sequences. Neighbor-joining method with 1000 bootstrap replications. *Phormidium* sp. LP904c is indicated in bold.

Table S1. Assignments, ion masses (*m*/*z*) and intensities (%) of the protonated non Arg microcystins of the most important product ions. Red No = Ion structure confirmed by Diehnelt et al. 2006 with high resolution fourier transform ion cyclotron resonance mass spectrometer. CI = Code for ion structure presented in Figure S5.

		Protonated product ion structures		MC-LTyr		MC-LHty		MC-HphHty		MC-LW		MC-LHph		MC-HphHph	
No	CI	[peptide + H]$^+$	-Neutral	m/z	Intensity	m/z	Intensity	m/z	Intensity	m/z	Intensity	m/z	Intensity	m/z	Intensity
1	1a-7c	Ala-Aa2-Asp-Aa4-Adda-Glu-Mdha + H		1 002		1 016		1 064		1 025		1 000		1 048	
2	1a-7c	Ala-Aa2-Asp-Aa4-Adda-Glu-Mdha + H	NH_3	985	100	999	100	1 047	85	1 008	100	983	92	1 031	100
3	1a-7c	Ala-Aa2-Asp-Aa4-Adda-Glu-Mdha + H	H_2O	984	80	998	38	1 046	35	1 007	53	982	70	1 030	62
4	1a-7c	Ala-Aa2-Asp-Aa4-Adda-Glu-Mdha + H	CH_3OH	970	9	984	6	1 032	6	993	8	968	10	1 016	14
5	1a-7c	Ala-Aa2-Asp-Aa4-Adda-Glu-Mdha + H	H_2O, NH_3	967	16	981	9	1 029	11	990	15	965	14	1 013	10
6	1a-7c	Ala-Aa2-Asp-Aa4-Adda-Glu-Mdha + H	CH_3OH, NH_3	953	14	967	11	1 015	10	976	16	951	13	999	11
7	1a-7c	Ala-Aa2-Asp-Aa4-Adda-Glu-Mdha + H	CH_3OH, H_2O	952	20	966	4	1 014	5	976	16	950	9	998	6
8	1a-6c	Ala-Aa2-Asp-Aa4-Adda-Glu + H	NH_3	902	10	916	11	964	8	925	9	900	5	948	7
9	1a-6c	Ala-Aa2-Asp-Aa4-Adda-Glu + H	H_2O	901	9	915	5	963	3	924	4	899	5	947	4
10	1a-4c	Ala-Aa2-Asp-Aa4 + H		477	24	491	20	539	22	500	30	475	20	523	25
11	2a-4c	Aa2-Asp-Aa4 + H		406	5	420	6	468	7	429	4	404	8	452	6
12	3a-7c	MeAsp-Aa4-Adda-Glu-Mdha + H		818	13	832	21	832	0	841	10	816	12	816	0
13	3a-7c	MeAsp-Aa4 + H		293	6	307	2	307	2	316	6	291	3	291	1
14	4a-7c	Aa4-Adda-Glu-Mdha + H		689	8	703	6	703	0	712	4	687	12	687	0
15	4a-5c	Aa4-Adda + H		477	24	491	20	491	2	500	30	475	20	475	0
16	5y	$C_{11}H_{17}NO$-Glu-Mdha-Ala-Aa2-Asp-Aa4 + H		868	8	882	56	930	60	891	48	866	100	914	84
17	5y-5x-4c	$C_{11}H_{14}O$-Glu-Mdha-Ala-Aa2-Asp-Aa4 + H		851	77	865	72	913	77	874	48	849	84	897	76
18	5a-5y-4c	$C_{11}H_{17}NO$-Glu-Mdha-Ala-Aa2-Asp-Aa4 + H	H_2O	850	63	864	31	912	36	873	31	848	69	896	38
19	5a-2c	Adda-Glu-Mdha-Ala-Aa2 + H	NH_3	693	35	693	15	741	12	693	23	693	29	741	14
20	5y-5x-3c	$C_{11}H_{14}O$-Glu-Mdha-Ala-Aa2-Asp + H		688	5	688	3	736	4	688	3	688	6	736	7
21	5a-1c	Adda-Glu-Mdha-Ala + H	NH_3	580	20	580	12	580	16	580	12	580	22	580	12
22	5y-5x-2c	$C_{11}H_{14}O$-Glu-Mdha-Ala-Aa2 + H		559	28	559	16	607	20	559	15	559	44	607	30
23	5a-7c	Adda-Glu-Mdha + H	NH_3	509	33	509	38	509	17	509	17	509	30	509	17
24	5y-5x-1c	$C_{11}H_{14}O$-Glu-Mdha-Ala + H		446	15	446	18	446	20	446	22	446	21	446	23
25	5y-5x-7c	$C_{11}H_{14}O$-Glu-Mdha + H		375	27	375	20	375	19	375	10	375	36	375	16
26	6a-4c	Glu-Mdha-Ala-Aa2-Asp-Aa4 + H		689	8	703	6	751	6	712	4	687	12	735	13
27	6a-3c	Glu-Mdha-Ala-Aa2-Asp + H	NH_3	509	33	509	38	557	22	509	17	509	30	557	1
28	6a-3c	Glu-Mdha-Ala-Aa2-Asp + H	H_2O	508	12	508	33	556	41	508	5	508	7	556	4
29	6a-2c	Glu-Mdha-Ala-Aa2 + H		397	6	397	5	445	7	397	5	397	8	445	4
30	7a-4c	Mdha-Ala-Aa2-Asp-Aa4 + H		560	51	574	44	622	40	583	42	558	58	606	48
31	7a-3c	Mdha-Ala-Aa2-Asp + H		397	6	397	5	445	7	397	5	397	8	445	4

Table S2. Assignments, ion masses (*m/z*) and intensities (%) of the sodiated non Arg microcystins of the most important product ions. CI = Code for ion structure

		Sodiated product ion structures		MC-LTyr		MC-LHty		MC-HphHty		MC-LW		MC-LHph		MC-HphHph	
No	CI	[peptide + Na]$^+$	- Neutral	m/z	Intensity	m/z	Intensity	m/z	Intensity	m/z	Intensity	m/z	Intensity	m/z	Intensity
1	1a-7c	Ala-Aa2-MeAsp-Aa4-Adda-Glu-Mdha		1 024		1 038		1 086		1 047		1 022		1 070	
2	1a-7c	Ala-Aa2-MeAsp-Aa4-Adda-Glu-Mdha	NH_3	1 007	9	1 021	11	1 069	11	1 030	17	1 005	11	1 053	7
3	1a-7c	Ala-Aa2-MeAsp-Aa4-Adda-Glu-Mdha	H_2O	1 006	14	1 020	12	1 068	16	1 029	16	1 004	13	1 052	16
4	1a-7c	Ala-Aa2-MeAsp-Aa4-Adda-Glu-Mdha	CO	996	57	1 010	52	1 058	59	1 019	46	994	48	1 042	47
5	1a-7c	Ala-Aa2-MeAsp-Aa4-Adda-Glu-Mdha	CO_2	980	7	994	10	1 042	11	1 003	6	978	8	1 026	9
6	1a-7c	Ala-Aa2-MeAsp-Aa4-Adda-Glu-Mdha	CO, H_2O	978	9	992	4	1 040	9	1 001	7	960	2	1 008	2
7	1a-7c	Ala-Aa2-MeAsp-Aa4-Adda-Glu-Mdha	CO_2, CO	952	37	966	28	1 014	29	975	26	950	23	998	28
8	1a-4c	Ala-Aa2-MeAsp-Aa4		499	6	513	6	561	3	522	11	497	3	545	6
9	1a-3c	Ala-Aa2-MeAsp	CO	308	5	308	4	356	8	308	2	308	6	356	18
10	2a-7c	Aa2-MeAsp-Aa4-Adda-Glu-Mdha		953	18	967	21	1 015	18	976	10	951	20	999	20
11	3a-1c	MeAsp-Aa4-Adda-Glu-Mdha-Ala		911	12	925	10	925	14	934	20	909	20	909	18
12	3a-6c	MeAsp-Aa4-Adda-Glu		757	11	771	10	771	9	780	5	755	4	755	6
13	3a-6c	MeAsp-Aa4-Adda-Glu	H_2O	739	5	753	8	753	8	762	13	737	8	737	5
14	3a-6c	MeAsp-Aa4-Adda-Glu	CO, H_2O	711	15	725	11	725	9	734	14	709	7	709	11
15	3a-5c	MeAsp-Aa4-Adda		628	42	642	39	642	34	651	46	626	46	626	46
16	3a-5c	MeAsp-Aa4-Adda	CO	600	53	614	59	614	43	623	53	598	42	598	37
17	4a-2c	Aa4-Adda-Glu-Mdha-Ala-Aa2		895	15	909	16	957	17	918	24	893	19	941	11
18	4a-2c	Aa4-Adda-Glu-Mdha-Ala-Aa2	CO	867	20	881	17	929	23	890	7	865	25	913	18
19	4a-7c	Aa4-Adda-Glu-Mdha		711	15	725	11	725	9	734	14	709	7	709	11
20	4a-6c	Aa4-Adda-Glu		628	42	642	39	642	34	651	46	626	46	626	46
21	4a-6c	Aa4-Adda-Glu	CO	600	53	614	59	614	43	623	53	598	42	598	37
22	4a-6c	Aa4-Adda-Glu	CO, H_2O	582	32	596	34	596	11	605	23	580	30	580	2
23	4a-5c	Aa4-Adda		499	6	513	6	513	6	522	11	497	3	497	2
24	5a-3c	Adda-Glu-Mdha-Ala-Aa2-MeAsp	NH_3	844	36	844	30	892	33	844	26	844	44	892	46
25	5a-7c	Adda-Glu-Mdha		548	7	548	9	548	<1	548	13	548	8	548	<1
26	6a-4c	Glu-Mdha-Ala-Aa2-MeAsp-Aa4		711	15	725	11	773	4	734	14	709	7	757	4
27	6a-3c	Glu-Mdha-Ala-Aa2-MeAsp		548	7	548	9	596	11	548	13	548	8	596	3
28	6a-3c	Glu-Mdha-Ala-Aa2-MeAsp	CO, H_2O	502	14	502	10	550	12	502	14	502	15	550	13
29	6a-2c	Glu-Mdha-Ala-Aa2		419	100	419	100	467	100	419	100	419	100	467	100
30	7a-5c	Mdha-Ala-Aa2-MeAsp-Aa4-Adda		895	15	909	16	957	17	918	24	893	19	941	11
31	7a-5c	Mdha-Ala-Aa2-MeAsp-Aa4-Adda	CO	867	20	881	17	929	23	890	7	865	25	913	21
32	7a-4c	Mdha-Ala-Aa2-MeAsp-Aa4		582	32	596	34	644	38	605	23	580	30	628	34
33	7a-3c	Mdha-Ala-Aa2-MeAsp		419	100	419	100	467	100	419	100	419	100	467	100
34	7a-3c	Mdha-Ala-Aa2-MeAsp	CO_2, CO	347	57	347	55	395	64	347	38	347	48	395	73

Table S3. Microcystin variants, retention times (R_t), relative amounts (RA) and small diagnostic ions (from protonated MC's) with corresponding amino acids from *Phormidium* sp. LP904c by HPLC-ITMS and UPLC-QTOF. RA's were calculated from the sum of the peak areas of the extracted ion chromatograms of different ion species (H, Na, K and 2H) of microcystins. Hty = homotyrosine, Hph = homophenylalanine. Diagnostic ions of MC's No 14-16 were analyzed from sodiated MC's and additionally MC No 15 ions masses were corrected with the mass of sodiated MC No 15 exact mass.

		**Aa in pos**		**Rt**		**Diagnostic ions (m/z) in MS^2**					
**No**	**Microcystin**	**X**	**Z**	**(min)**	**RA (%)**	**Aa^2**		**Aa^4**		**Mdha^7**	**Glu^6-Mdha^7**
1	MC-YR	Y	R	35,5	<1						
2	MC-HtyR	Hty	R	36,3	4	150,0913	Hty	70,0649	R	56,0489	213,0868
3	MC-MR	M	R	36,5	1						
4	[DMAdda^5]MC-LR	L	R	36,8	<1						
5	[MeSer^7]MC-LR	L	R	38,2	<1						
6	[Asp^3]MC-LR	L	R	38,3	<1						
7	MC-LR	L	R	38,8	55	86,0959	L	70,0649	R	56,0492	213,0867
8	MC-FR	F	R	38,8	9	120,0809	F	70,0653	R	56,0499	213,0864
9	[Dha^7]MC-LR	L	R	39,3	<1						
10	MC-HphR	Hph	R	41,2	17	134,0959	Hph	70,0645	R	56,0483	213,0869
11	MC-LY	L	Y	44,9	1						
12	MC-LHty	L	Hty	45,1	5	86,0957	L	150,0909	Hty	56,0489	213,0859
13	MC-HphHty	Hph	Hty	46,9	3	134,0959	Hph	150,0911	Hty	56,0487	213,0864
14	MC-LW	L	W	47,6	<1	86,0970	L	159,0873	W	56,0503	213,0877
15	MC-LHph	L	Hph	51,2	<1	86,0979	L	134,0950	Hph	-	213,0856
16	MC-HphHph	Hph	Hph	52,9	<1	-		-		56,0471	213,0870

Table S4. Sequence similarity of the microcystin gene cluster from *Phormidium* sp. LP904c obtained by BLASTp or Blastx.

**Amino acid sequence from *Phormidium* sp. LP904c**	**Query length (amino acids)**	**Predicted function**	**Organism**	**Coverage/Identity**	**Accession number**
McyA	2783	NRPS	*Fischerella* sp. PCC 9339	99/75	WP_017308558.1
			Dolichospermum/Anabaena sp. 90	100/75	WP_041458258.1
McyB	2130	NRPS	*Fischerella* sp. PCC 9339	99/76	WP_017308559.1
			Dolichospermum/Anabaena sp. 90	99/76	WP_015078940.1
McyC	1296	NRPS	MULTISPECIES: *Planktothrix*	99/80	WP_026787621.1
			Planktothrix agardhii	99/80	WP_042154169.1
McyD	3907	PKS	*Planktothrix prolifica* NIVA-CYA98	100/78	WP_026796481.1
			*Planktothrix agardhi*i NIVA-CYA126/8	100/78	WP_042154180.1
McyE	3489	NRPS-PKS	*Planktothrix agardhii* NIVA-CYA56/3	100/82	WP_027255262.1
			MULTISPECIES: *Planktothrix*	100/82	WP_026795534.1
McyF	238	Aspartate racemase	*Microcystis aeruginosa* PCC9807	97/83	WP_002768078.1
			Microcystis aeruginosa PCC7941	97/83	WP_002776433.1
McyG	2644	NRPS-PKS	*Planktothrix prolifica* NIVA/CYA98	100/82	WP_026796479.1
			Planktothrix agardhii NIVA/CYA126/8	100/82	WP_042154175.1
McyH	584	ABC transporter	*Planktothrix agardhii*	99/82	WP_027250110.1
			MULTISPECIES: *Planktothrix*	99/81	WP_026787624.1
McyI	336	Dehydrogenase	*Microcystis aeruginosa* NIES88	99/77	WP_061431771.1
			Microcystis aeruginosa PCC9808	99/76	WP_044034182.1
McyJ	316	Methyltransferase	MULTISPECIES: *Planktothrix*	97/87	WP_026795539.1
			Planktothrix agardhii NIVA-CYA56/3	97/86	WP_027255267.1

Table S5. Microcystins variants detected in the strains from table 2 and 3. In highlight are the homoamino acids detected.

Genera	Strain	Microcystin variants	Amino acids in microcystin structure correspondent to the A domain		% MC*	Ref.¤
			McyB1 (X)	McyC (Y)		
Phormidium sp.	DVL1003c	MC-LR	Leu	Arg	49	1, 2
		MC-HphR	Hph	-	14	
		MC-LHty	-	-	12	
		MC-HphHty	Hph	Hty	7	
		MC-FR	Phe	-	7	
		MC-HtyR	Hty	-	4	
		[MeSer⁷]MC-LR	-	-	2	
		[D-Asp³]MC-LHty	-	Hty	2	
		MC-LHph	-	Hph	1	
		MC-HphHph	Hph	Hph	<1	
		MC-LW	-	Trp	<1	
		[D-Asp³]MC-LR	-	-	<1	
		[Dha⁷]MC-LR	-	-	<1	
		[DMAdda⁵]MC-LR	-	-	<1	
		MC-YR	Tyr	-	<1	
Planktothrix agardhii	CYA 126/8	[D-Asp³]MC-RR	Arg	-	92	3
		[D-Asp³]MC-LR	-	-	7	
Planktothrix agardhii	213	[D-Asp³]MC-RR	Arg	-	84	4
		[D-Asp³]MC-LR	-	-	15	
		[Asp³, Dha⁷]MC-LR	-	-	<1	
Planktothrix agardhii	NIVA-CYA56/3	Dm-MC-LR	-	-	Ni	5
		Dm-MC-RR	Arg	-	Ni	
		Dm-MC-YR	Tyr	-	Ni	
Planktothrix prolifica	NIVA-CYA 98	[Dha⁷]MC-LR	-	-	Ni	6
		[Dha⁷]MC-RR	Arg	-	Ni	
Planktothrix rubescens	NIVA-CYA 407	Dm-MC-LR	-	-	Ni	5

		Dm-MC-RR	Arg	-	Ni	
		Dm-MC-HtyR	Hty	-	Ni	
Dolichospermum sp.	90	MC-LR	-	-	55	4
		[D-Asp^3]MC-LR	-	-	34	
		MC-RR	Arg	-	5	
		[D-Asp^3]MC-RR	Arg	-	2	
		MC-HilR	Hil	-	1.5	
		[D-Asp^3]MC-HilR	Hil	-	<1	
		[MeSer^7]MC-LR	-	-	<1	
		[DMAdda^5]MC-LR	-	-	<1	
		[D-Asp^3, MeSer^7]MC-LR	-	-	<1	
		[Dha^7]MC-LR	-	-	<1	
Dolichospermum flos-aquae	18B6	[D-Asp^3, Dha^7]MC-RR	Arg	-	79	4
		Demethyl- MC-RR	Arg	-	20	
		[X]MC-RR	Arg	-	1	
		MC-XR	X	-	<1	
Dolichospermum lemmermannii	66 A	[Dha^7]MC-HtyR	Hty	-	56	4
		[D-Asp^3, Dha^7]MC-XR	X	-	29	
		[L-Ser^7]MC-HtyR	Hty	-	4	
		[D-Asp^3, L-Ser^7]MC-HtyR	Hty	-	2	
		[Dha^7]MC-LR	-	-	2	
		[Dha^7]MC-HphR	Hph	-	2	
		[D-Asp^3, Dha^7]MC-LR	-	-	1	
		[DMAdda^5, (M)dha^7]MC-(H)tyR	Hty/Tyr	-	<1	
		[D-Asp^3, Dha^7]MC-XR	X	-	<1	
		[X]MC-HtyR	Hty	-	<1	
		[D-Asp^3]MC-XR	X	-	<1	
		[X]MC-(H)tyR	Hty/Tyr	-	<1	
		MC-XR	X	-	<1	
		[X]MC-X/HtyR	X/Hty	-	<1	
		[X]MC-(H)tyR	Hty/Tyr	-	<1	
		MC-HtyR	Hty	-	<1	

		[X, L-Ser[7]]MC-LR	-	-	<1	
		[D-Asp[3]]MC-XR	X	-	<1	
		Demethyl-[L-Ser[7]]MC-LR	-	-	<1	
		[L-Ser[7]]MC-LR	-	-	<1	
		[Dha[7]]MC-FR	Phe	-	<1	
		[D-Asp[3], Dha[7]]MC-FR	Phe	-	<1	
		[X]MC-LR	-	-	<1	
		[D-Asp[3], Dha[7]]MC-HphR	Hph	-	<1	
Nostoc sp.	152	[ADMAdda[5]]MC-LR	-	-	44	4, 7
		[ADMAdda[5]]MC-LHar	-	Har	40	
		[ADMAdda[5]]MC-HilR	Hil	-	1.7	
		[ADMAdda[5]]MC-HilHar	Hil	Har	1	
		[ADMAdda[5], Dha[7]]MC-LR	-	-	<1	
		[Ser[1], D-Asp[3],ADMAdda[5]]MC-LR	-	-	<1	
		[D-Asp[3],ADMAdda[5], Dha[7]]MC-LR	-	-	<1	
		[DMAdda[5]]MC-LR	-	-	<1	
		[DMAdda[5]]MC-LHar	-	Har	<1	
		[D-Asp3, DMAdda[5]]MC-LR	-	-	<1	
		[ADMAdda[5]]MC-XR	X	-	<1	
		[D-Asp[3], ADMAdda[5]]MC-VR	Val	-	<1	
		[D-Asp[3], ADMAdda[5], Dha[7]]MC-HilR	Hil	-	<1	
Nostoc sp.	IO-102-I	[ADMAdda[5]]MC-LR	-	-	82	4, 8
		[ADMAdda[5]]MC-XR	X	-	4	
		[D-Asp[3], ADMAdda[5]]MC-LR	-	-	3	
		[DMAdda[5]]MC-LR	-	-	2	
		MC-XR	X	-	1	
		MC-XR	X	-	1	
		[(X), ADMAdda[5]]MC-(F)R	Phe	-	1	
		MC-XR	X	-	1	
		[(X), DMAdda[5]]MC-(Hil)R	Hil	-	1	
		[DMAdda[5]]MC-HilR	Hil	-	<1	
		[ADMAdda[5]]MC-XR	X	-	<1	
		[ADMAdda[5]]MC-XR	X	-	<1	
		[ADMAdda[5]]MC-YR	Tyr	-	<1	
		[ADMAdda[5]]MC-XR	X	-	<1	
		[X]MC-LR	-	-	<1	
		MC-XR	X	-	<1	

		[X]MC-LR	-	-	<1	
		MC-XR	X	-	<1	
Fischerella sp.	PCC9339	MC-LR	-	-	Ni	9
Hapalosiphon hibernicus	BZ-3-1	MC-LA	-	Ala	70	4
		[D-Asp^{3}]MC-LA	-	Ala	16	
		MC-RA	Arg	Ala	7	
		[D-Asp^{3}]MC-RA	Arg	Ala	2	
		MC-VA	Val	Ala	1	
		[D-Asp^{3}, DMAdda^{5}]MC-LA	-	Ala	1	
		[Dha^{7}]MC-LA	-	Ala	1	
		MC-LL	-	Leu	<1	
		[D-Asp^{3}]MC-VA	Val	Ala	<1	
		[D-Asp^{3}]MC-LV	-	Val	<1	
		MC-LV	-	Val	<1	
Microcystis aeruginosa	PCC 7806	[D-Asp^{3}]MC-LR	-	-	52	4
		MC-LR	-	-	46	
		[MeSer^{7}]MC-LR	-	-	<1	
		[D-Asp^{3}, Dha^{7}]MC-LR	-	-	<1	
		[Dha^{7}]-MC-LR	-	-	<1	
		[D-Asp^{3}, MeSer^{7}]MC-LR	-	-	<1	
Microcystis aeruginosa	FCY-28	Ni	Ni	Ni	Ni	10
Microcystis aeruginosa	FCY-26	Ni	Ni	Ni	Ni	10
Microcystis aeruginosa	UV027	MC-RR	Arg	-	100	11
Microcystis aeruginosa	K139	[Dha^{7}]MC-LR	-	-	76	12
		[Asp^{3}, Dha^{7}]MC-LR	-	-	24	
Microcystis aeruginosa	DIANCHI905	Ni	Ni	Ni	Ni	13
Microcystis aeruginosa	PCC 9807	Ni	Ni	Ni	Ni	14
Microcystis aeruginosa	PCC 7941	Ni	Ni	Ni	Ni	14

Microcystis aeruginosa	PCC 9443	Ni	Ni	Ni	Ni	14
Microcystis aeruginosa	NIES-843	MC-RR	Arg	-	50	15
		MC-LR	-	-	41	
		MC-YR	Tyr	-	10	
Microcystis aeruginosa	SPC777	[L-Ser^{7}] MC-RR	Arg	-	100	16
Microcystis viridis	NIES 102	MC-HilR	Hil	-	19	4
		[Adda/DMAdda^{5}]MC-LR	Leu/Hil	-	15	
		MC-RR	Arg	-	10	
		MC-YR	Tyr	-	9	
		[X, D-Asp^{3}]MC-HilR	Hil	-	6	
		MC-WR	Trp	-	5	
		[D-Asp^{3}]MC-LR	-	-	2	
		[D-Asp^{3}]MC-YR	Tyr	-	2	
		MC-FR	Phe	-	2	
		MC-HtyR	Hty	-	2	
		MC-XR	X	-	2	
		MC-XR	X	-	2	
		MC-XR	X	-	1	
		[D-Asp^{3}]MC-WR	Trp	-	<1	
		[D-Asp^{3}]MC-RR	Arg	-	<1	
		[D-Asp^{3}]MC-XR	X	-	<1	
		[Ser^{1}, D-Asp^{3}, Dha^{7}]MC-LR	-	-	<1	
		[X, MeSer^{7}]MC-(Hil)R	Hil	-	<1	
		[Dha^{7}]MC-LR	-	-	<1	
		[X, D-Asp^{3}]MC-HilR	Hil	-	<1	
		[D-Asp^{3}]MC-XR	X	-	<1	
		MC-XR	X	-	<1	
		MC-XR	X	-	<1	
		MC-XR	X	-	<1	
		MC-XR	X	-	<1	
		MC-XR	X	-	<1	
		MC-XR	X	-	<1	
		MC-XR	X	-	<1	
		MC-XR	X	-	<1	
		MC-XR	X	-	<1	

MC-XR	X	-	<1
MC-XR	X	-	<1
MC-XR	X	-	<1
MC-XR	X	-	<1
MC-XR	X	-	<1
MC-XR	X	-	<1
MC-XR	X	-	<1
MC-XR	X	-	<1
MC-XR	X	-	<1
MC-XR	X	-	<1
MC-XR	X	-	<1
MC-XR	X	-	<1
MC-XR	X	-	<1
MC-XR	X	-	<1

X. MC contains an unknown amino acid or the overall amino acid content is not known. * % MC. Amounts of microcystin detected. Ni. No information.

¤ References:

1. Shishido TK, Kaasalainen U, Fewer DP, Rouhiainen L, Jokela J, Wahlsten M, Fiore MF, Yunes JS, Rikkinen J, Sivonen K. 2013. Convergent evolution of [D-Leucine[1]] microcystin-LR in taxonomically disparate cyanobacteria. *BMC Evol. Biol.* 13:86.
2. Izaguirre G, Jungblut AD, Neilan BA. 2007. Benthic cyanobacteria (Oscillatoriaceae) that produce microcystin-LR, isolated from four reservoirs in southern California. Water Res. 41(2):492-498.
3. Christiansen G, Molitor C, Philmus B, Kurmayer R. 2008. Nontoxic strains of cyanobacteria are the result of major gene deletion events induced by a transposable element. Mol. Biol. Evol. 25(8):1695–1704.
4. Fewer DP**,** Rouhiainen L**,** Jokela J**,** Wahlsten M**,** Laakso K**,** Wang H**,** Sivonen K**.** 2007. Recurrent adenylation domain replacement in the microcystin synthetase gene cluster. *BMC Evol Biol.* **7:**183.
5. Tooming-Klunderud A, Sogge H, Rounge TB, Nederbragt AJ, Lagesen K, Glöckner G, Hayes PK, Rohrlack T, Jakobsen KS. 2013. From green to red: horizontal gene transfer of the phycoerythrin gene cluster between *Planktothrix* strains. *Appl Environ Microbiol*. 79(21): 6803–6812.
6. Rounge TB, Rohrlack T, Nederbragt AJ, Kristensen T, Jacobsen KS. 2009. A genome-wide analysis of nonribosomal peptide synthetase gene clusters and their peptides in a *Planktothrix rubescens* strain. BMC Genomics. 10:396.
7. Sivonen, K, Namikoshi M, Evans WR, Fardig M, Carmichael WW, Rinehart KL. 1992. Three new microcystins, cyclic heptapeptide hepatotoxins, from *Nostoc* sp. strain 152. Chem. Res. Toxicol. 5:464–469.
8. Oksanen I, Jokela J, Fewer DP, Wahlsten M, Rikkinen J, Sivonen K. 2004. Discovery of rare and highly toxic microcystins from lichen-associated cyanobacterium *Nostoc* sp. strain IO-102-I. Appl Environ Microbiol. 70(10): 5756–5763.

9. Calteau A, Fewer DP, Latifi A, Coursin T, Laurent T, Jokela J, Kerfeld CA, Sivonen K, Piel J, Gugger M. 2014. Phylum-wide comparative genomics unravel the diversity of secondary metabolism in Cyanobacteria. *BMC Genomics.* 15: 977.
10. Rhee J-S, Dahms H-U, Choi B-S, Lee J-S, Choi I-Y. 2012. Identification and analysis of whole microcystin synthetase genes from two Korean strains of the cyanobacterium *Microcystis aeruginosa.* Genes & Genomics. 34(4), 435-439.
11. Tooming-Klunderud A, Fewer DP, Rohrlack T, Jokela J, Rouhiainen L, Sivonen K, Kristensen T, Jakobsen KS. 2008. Evidence for positive selection acting on microcystin synthetase adenylation domains in three cyanobacterial genera. BMC Evol Biol. 8:256.
12. Harada K-I, Ogawa K, Matsuura K, Nagai H, Murata H, Suzuki M, Itezono Y, Nakayama N, Shirai M, Nakano M. 1991. Isolation of two toxic heptapeptide microcystins from an axenic strain of *Microcystis aeruginosa*, K-139. Toxicon 29: 479–489.
13. Yang C, Lin F, Li Q, Li T, Zhao J. 2015. Comparative genomics reveals diversified CRISPR-Cas systems of globally distributed *Microcystis aeruginosa*, a freshwater bloom-forming cyanobacterium. Front Microbiol. 2015; 6: 394.
14. Humbert J-F, Barbe V, Latifi A, Gugger M, Calteau A, Coursin T, Lajus A, Castelli V, Oztas S, Samson G, Longin C, Medigue C, de Marsac NT. 2013. A tribute to disorder in the genome of the bloom-forming freshwater cyanobacterium *Microcystis aeruginosa*. PLoS One. 8: e70747.
15. Srivastava A, Choi GG, Ahn CY, Oh HM, Ravi AK, Asthana RK. 2012. Dynamics of microcystin production and quantification of potentially toxigenic *Microcystis* sp. using real-time PCR. Water Res. 46:817-27.
16. Sant'Anna CL, de Carvalho LR, Fiore MF, Silva-Stenico ME, Lorenzi AS, Rios FR, Konno K, Garcia C, Lagos N. 2011. Highly toxic *Microcystis aeruginosa* strain, isolated from São Paulo-Brazil, produce hepatotoxins and paralytic shellfish poison neurotoxins. Neurotox Res. 19(3):389-402.
